# Genetic Selection for Context-Dependent Stochastic Phenotypes: Sp1 and TATA Mutations Increase Phenotypic Noise in HIV-1 Gene Expression

**DOI:** 10.1371/journal.pcbi.1003135

**Published:** 2013-07-11

**Authors:** Kathryn Miller-Jensen, Ron Skupsky, Priya S. Shah, Adam P. Arkin, David V. Schaffer

**Affiliations:** 1 Department of Biomedical Engineering, Yale University, New Haven, Connecticut, United States of America; 2 California Institute for Quantitative Biosciences, University of California, Berkeley, Berkeley, California, United States of America; 3 Department of Bioengineering, University of California, Berkeley, California, United States of America; 4 Physical Biosciences Division, Lawrence Berkeley National Laboratory, Berkeley, California, United States of America; 5 Department of Chemical and Biomolecular Engineering, University of California, Berkeley, California, United States of America; Stanford University, United States of America

## Abstract

The sequence of a promoter within a genome does not uniquely determine gene expression levels and their variability; rather, promoter sequence can additionally interact with its location in the genome, or genomic context, to shape eukaryotic gene expression. Retroviruses, such as human immunodeficiency virus-1 (HIV), integrate their genomes into those of their host and thereby provide a biomedically-relevant model system to quantitatively explore the relationship between promoter sequence, genomic context, and noise-driven variability on viral gene expression. Using an *in vitro* model of the HIV Tat-mediated positive-feedback loop, we previously demonstrated that fluctuations in viral Tat-transactivating protein levels generate integration-site-dependent, stochastically-driven phenotypes, in which infected cells randomly ‘switch’ between high and low expressing states in a manner that may be related to viral latency. Here we extended this model and designed a forward genetic screen to systematically identify genetic elements in the HIV LTR promoter that modulate the fraction of genomic integrations that specify ‘Switching’ phenotypes. Our screen identified mutations in core promoter regions, including Sp1 and TATA transcription factor binding sites, which increased the Switching fraction several fold. By integrating single-cell experiments with computational modeling, we further investigated the mechanism of Switching-fraction enhancement for a selected Sp1 mutation. Our experimental observations demonstrated that the Sp1 mutation both impaired Tat-transactivated expression and also altered basal expression in the absence of Tat. Computational analysis demonstrated that the observed change in basal expression could contribute significantly to the observed increase in viral integrations that specify a Switching phenotype, provided that the selected mutation affected Tat-mediated noise amplification differentially across genomic contexts. Our study thus demonstrates a methodology to identify and characterize promoter elements that affect the distribution of stochastic phenotypes over genomic contexts, and advances our understanding of how promoter mutations may control the frequency of latent HIV infection.

## Author Summary

The sequence of a gene within a cellular genome does not uniquely determine its expression level, even for a single type of cell under fixed conditions. Numerous other factors, including gene location on the chromosome and random gene-expression “noise,” can alter expression patterns and cause differences between otherwise identical cells. This poses new challenges for characterizing the genotype–phenotype relationship. Infection by the human immunodeficiency virus-1 (HIV-1) provides a biomedically important example in which transcriptional noise and viral genomic location impact the decision between viral replication and latency, a quiescent but reversible state that cannot be eliminated by anti-viral therapies. Here, we designed a forward genetic screen to systematically identify mutations in the HIV promoter that alter the fraction of genomic integrations that specify noisy/reactivating expression phenotypes. The mechanisms by which the selected mutations specify the observed phenotypic enrichments are investigated through a combination of single-cell experiments and computational modeling. Our study provides a framework for identifying genetic sequences that alter the distribution of stochastic expression phenotypes over genomic locations and for characterizing their mechanisms of regulation. Our results also may yield further insights into the mechanisms by which HIV sequence evolution can alter the propensity for latent infections.

## Introduction

Non-genetic heterogeneity is a ubiquitous feature of cellular gene expression that can significantly impact the genotype–phenotype relationship. Even under highly controlled culture conditions, a clonal population of cells may demonstrate a broad range of expression levels for a given gene [1]–[4]. At least some of this variability, often termed ‘noise’, is believed to arise from the intrinsically stochastic nature of the biochemical processes involved in gene expression [5], [6]. Studies that couple quantitative experimentation with mathematical modeling have begun to reveal the mechanisms by which non-genetic variability is generated and moderated [7], finding that noise: differentially impacts the expression of functional classes of genes [8], [9]; can be propagated, amplified, or attenuated by gene regulatory circuits [10], [11]; and is subject to selective pressure [12]–[15]. Stochastically-generated expression variability is increasingly appreciated to have important phenotypic consequences in diverse cellular settings, including bacterial evasion of antibiotic treatment [16], multi-cellular development [17], cancer development and progression [18], and viral latency [19], [20].

Recent evidence demonstrates that the chromosomal position of a gene, or its genomic context, affects both its mean expression level and expression noise [21]–[24]. One mechanism by which genomic context modulates gene expression is by specifying the dynamics of the local chromatin state, which can impact multiple neighboring genes [3], [25], [26]. Additionally, endogenous genes can sample different genomic environments through translocation and recombination, impacting diverse biological processes including species evolution, organism development, and cancer [27], [28]. Human retroviruses, such as human immunodeficiency virus-1 (HIV), also sample genomic environments through semi-random integration into the host genome, which in turn affects viral replication [29]. Thus, genomic context impacts cellular phenotypes and offers additional dimensions of selectable variation that shape the architecture and evolution of eukaryotic genomes, as well as the retroviruses that invade them.

Stochastic gene expression phenotypes that are modulated by genomic context present new challenges for quantifying the genotype–phenotype relationship. In particular, understanding how genomic context and gene sequence cooperate to alter gene expression dynamics requires quantifying how the sequences of regulatory elements alter the distribution of expression phenotypes over the set of genomic environments sampled by a gene. Gene regulatory networks may further alter gene expression phenotypes by amplifying or minimizing noise in gene expression through positive and negative feedback. Thus, when a genetic mutation is linked to a change in the distribution of stochastic phenotypes over genomic contexts, a further challenge is to identify the underlying mechanism that drives this change.

In this study, we identify promoter mutations that modulate context-dependent stochastic phenotypes in a lentiviral human immunodeficiency virus-1 (HIV) model system and investigate the mechanisms by which they impact viral gene expression. HIV exhibits a high degree of genetic variability due to its high replication rates [30] and the error-prone nature of reverse transcription [31], [32]. Following semi-random integration into the genome of host CD4+ T cells [29], HIV usually establishes a productive infection, but in rare cases can adopt a non-replicating but reversible latent phenotype, such as when an infected activated T cell transitions to a memory T cell [33], [34]. Latently infected cells do not express virus and thus cannot be effectively targeted by current therapeutics [35]; however, latent HIV can reactivate after long delays, leading to renewed viral spread [36]. Consequently, latent infection represents the single greatest obstacle to fully eradicating HIV in patients [37]. Importantly, a number of studies have demonstrated that genomic context and non-genetic variability play important roles in determining the replication-versus-latency decision of integrated HIV within a cell [19], [21], [22], [26]. Thus, HIV provides an ideal system for studying the interplay between gene sequence, genomic environment, and stochastic gene expression.

The virally encoded transcriptional activator Tat plays an essential role in HIV expression dynamics and the replication-versus-latency decision. The nascent HIV transcript forms a RNA hairpin, termed the HIV transactivation response element (TAR loop), that causes RNA polymerase II (RNAPII) to stall [38]. Tat binds to the TAR loop and in turn recruits the positive elongation factor b (p-TEFb), which phosphorylates RNAPII to relieve the stall and complete a cycle of transcription [39]. Transcript processing and translation then results in production of viral proteins, including more Tat. Thus, Tat enhances HIV transcriptional efficiency in a strong positive-feedback loop [40] that is necessary for viral gene expression from proviruses that immediately initiate replication or from latent infections that reactivate [41], [42].

We have previously demonstrated that an *in vitro* model of the HIV Tat positive feedback loop can generate a diverse range of stochastic phenotypes by sampling genomic contexts. These stochastic phenotypes include bimodal expression behaviors where non-expressing and highly expressing cells co-exist in a single clonal population [20], [43] and random switching between these two expression states occurs with significant delays. Noise in basal viral gene expression in the absence of Tat varies systematically over genomic integrations [21], [22], and its amplification by Tat feedback provides a possible mechanism to explain the diverse phenotypes generated in the presence of Tat. We have hypothesized that stochastically-driven delays in activation for some viral integrations are an intrinsic property of Tat positive feedback, and that these delays may provide a sufficient time window to establish latent infections *in vivo* when coupled to host-cell dynamics such as the transition to a memory T cell [20], [43]. Thus, HIV sequence mutations that affect the frequency of stochastic phenotypes *in vitro* may affect the frequency of latent infections *in vivo*. While isolated examples of promoter mutations that control context-dependent stochastic phenotypes have been investigated for HIV [43], no study has yet systematically identified such mutations or analyzed the mechanisms by which the distribution of phenotypes is modulated.

Here, we designed a forward genetic screen to select for HIV promoter mutations that increase the fraction of genomic integrations that result in stochastic gene expression phenotypes. Our screen identified important mutations in a number of core promoter regions, including Sp1 and TATA transcription factor binding sites. Through single-cell experiments, we confirmed that our strongest hits – point mutations in Sp1 site III and in the TATA box – increased the frequency of stochastic phenotypes several fold. We further demonstrated experimentally that the Sp1 mutation altered basal expression dynamics in the absence of Tat, and also impaired transactivated gene expression in the presence of Tat. Computational analysis demonstrated that the changes in basal expression observed for the Sp1 mutant could contribute significantly to the enrichment in stochastic phenotypes in the presence of impaired Tat feedback, if the mutation affected Tat-mediated amplification differentially across genomic contexts. Our analysis thus demonstrates a methodology for identifying genetic elements that affect the distribution of context-dependent stochastic phenotypes and the mechanisms by which they function. Our findings may also contribute to understanding how mutational selection could alter the frequency of latent HIV infection.

## Results

### Quantifying context-dependent stochastic phenotypes in an *in vitro* model of HIV-1 infection

**Figure 1:**
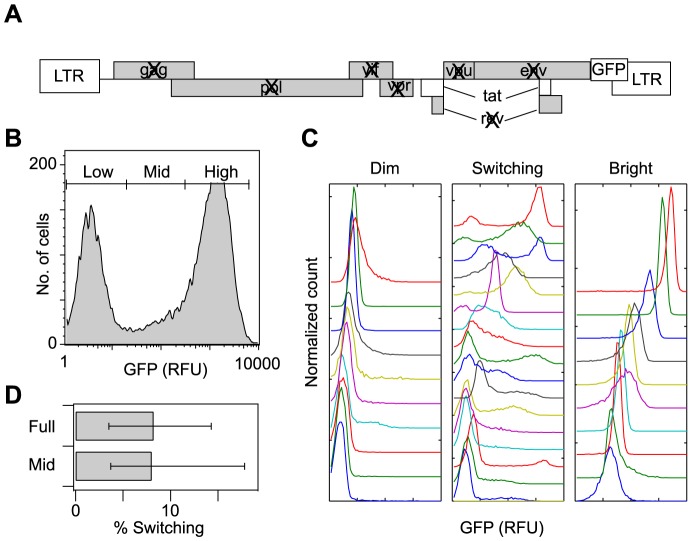
An *in vitro* model of HIV gene expression exhibits a distribution of integration-site-dependent phenotypes, including noise-driven Switching phenotypes. (A) Schematic of the full-length HIV lentiviral model of the Tat-mediated positive feedback loop (sLTR-Tat-GFP). Viral proteins other than Tat were inactivated and Nef was replaced with GFP. (B–C) Flow cytometry histogram of Jurkat cells infected with a single HIV WT virus for (B) a bulk population with mixed integration positions and (C) sample Jurkat clonal populations, each containing a single (different) genomic integration of the WT HIV provirus. Representative Dim and Bright clonal histograms were chosen to span the range of fluorescence means. For Switching phenotypes, representative clonal histograms were chosen from the distribution clusters that were used to define a quantitative Switching criterion. GFP axis range is the same for all histograms. (D) Quantification of the WT Switching fraction based on a stratified sample of clones from the full range of GFP expression (“Full”), and based on a sub-sample of clones sorted from only the Mid region of the bulk fluorescence range (“Mid”). Error bars mark 95% confidence intervals, estimated by a bootstrap method.

To quantitatively study stochastic gene expression of HIV infections as a function of genomic context, we adapted a full-length HIV NL4-3-based LTR lentiviral packaging platform [44] by introducing stop codons into all viral proteins except Tat and by replacing Nef with GFP (sLTR-Tat-GFP; Figure 1A). This minimal viral system, referred to in this study as wild type (WT), is similar to a model vector used previously in which Tat and GFP are expressed from a bicistronic lentiviral vector under control of the same LTR promoter [20], [43]. However, the new sLTR-Tat-GFP vector more closely mimics HIV gene expression, with Tat produced as a splice product of two exons as in natural HIV infection. The leukemic Jurkat T cell line was infected with sLTR-Tat-GFP at a low multiplicity of infection (MOI<0.1), such that the majority of infected cells (>95%) contained a single integrated provirus. The infected, GFP+ cells were then isolated by fluorescence activated cell sorting (FACS) after stimulation with tumor necrosis factor-α (TNFα) and cultured for ten days so that the population relaxed to a steady-state GFP expression profile. The resulting polyclonal or “bulk-infected” cell population showed bimodal gene expression, which indicated the presence and absence of Tat positive feedback in different cellular infections (Figure 1B), as observed with the previously studied bicistronic lentiviral vector [20], [43].

Bimodal Tat–GFP expression in the bulk-infected population arises from a mixture of integration events that result in either high or low gene expression, as well as individual integrations that result in variable or stochastic gene expression. To separate these contributions to the overall bulk distribution, we sorted individual cells – each containing a single (different) genomic integration of the provirus – from low, mid, or high ranges of GFP expression (Figure 1B). We then expanded these individual sorted cells to yield 125 single-integration clonal populations and subsequently quantified their GFP expression phenotypes by flow cytometry. Consistent with earlier studies [20], [43], a diverse spectrum of clonal GFP expression phenotypes was observed, including narrow single peaks of low or high GFP expression (referred to here as Dim and Bright distributions, respectively), as well as wide and/or bimodal distributions (Figure 1C). The wide/bimodal clonal distributions occurred with higher frequency within populations sorted from the mid-GFP range (Figure S1) and included both cells that are Bright, representing Tat-transactivated expression that would support viral replication, and cells that are Dim, representing low levels of basal expression that may be related to viral latency. Analogously, earlier work showed that when Dim cells are sorted from the bulk multi-integration population, a fraction eventually activated and migrated into the Bright range, and vice-versa [20], [22], [43]. We collectively refer to these stochastic viral gene expression phenotypes as “Switching” and consider them to be a model for latent infections that can randomly “switch” from an inactive state to a productive state.

Given HIV's rapid mutation rate [30]–[32], an interesting question is how changes in the viral promoter could affect the relative frequency of different expression phenotypes over the set of genomic environments that are sampled through infection and viral integration, and in particular whether specific mutations could increase the frequency of Switching phenotypes. As a first step in addressing this question, we developed objective, feature-based clustering criteria to classify gene expression behavior for a clonal population as Switching, Dim, or Bright. In this classification, cut-off values were manually selected for nine GFP-distribution measures that reflect expression heterogeneity, such as bimodality, width, and skewness (Table S1 and Figure S2). Distributions with a value exceeding the cut-off for any one of these features were labeled as Switching (details of methods described in Text S1). By applying these criteria uniformly to our initial collection of single-integration clones (Figure 1C), we estimated the fraction of integrations in our system that led to a Switching phenotype to be 8.2% (Figure 1D). We developed an alternate estimate of the Switching fraction based on sampling single-integration clones sorted only from the mid-GFP range and extrapolating to the full population (see Text S1). This method resulted in a similar Switching fraction estimate of 8% (Figure 1D), and was thus used in the remainder of our study for increased experimental efficiency.

### A stochastic model of Tat positive feedback demonstrates delayed activation for Switching phenotypes

**Figure 2:**
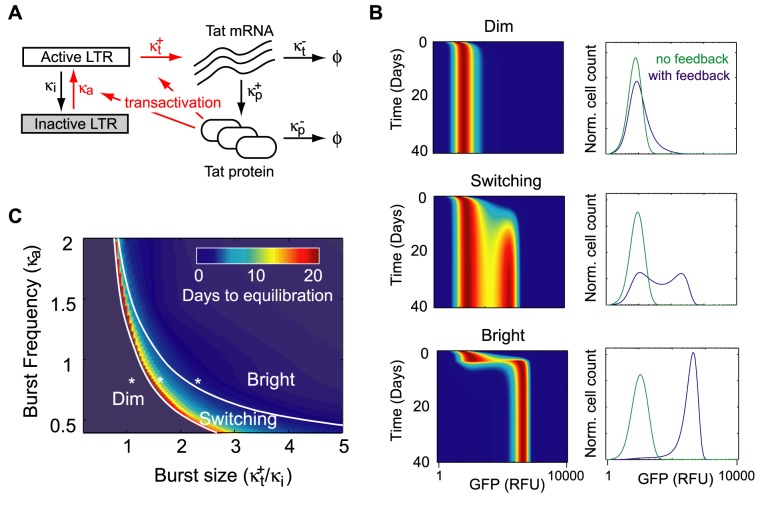
A computational model of LTR transcription with Tat feedback demonstrates noise-driven Switching phenotypes with delayed activation/deactivation (A) Model schematic: The viral LTR promoter probabilistically switches between a transcriptionally inactive state and a transcriptionally active state, with rates 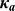 and 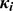. In the active state, transcripts are produced with rate 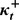, and degraded at rate 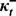. Protein translation occurs from each transcript independently at rate 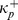, and each protein is degraded with rate 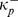. As a model of basal transcription, all rates are assumed constant, and transcript is produced in bursts when 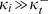 and 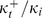 is of order 1 or greater [22]. For the transactivation circuit, the translated protein is Tat (plus GFP), and we include a Michaelis-Menten-like dependence on Tat for the promoter activation and the transcription rates (highlighted in red in the model schematic): 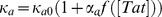, 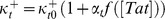, 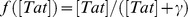. The parameters 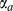 and 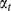 specify fold-amplification at saturated Tat binding, and 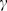 specifies the saturation concentration. The model output is the predicted steady-state distribution of protein (GFP and Tat) count across a clonal population of cells, which is then converted to cytometer RFU based on previous calibration [22]. (B) Simulated protein distributions were evolved over time from a Dim initialization (left) for representative parameter values that lead to Dim, Switching, and Bright steady-state phenotypes (right, blue curves). Simulated steady-state basal expression distributions for the same parameter values without Tat feedback are given for comparison (i.e. 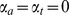; green curves). Simulated histograms are normalized and plotted on the same fluorescence axis as the cytometer data in Figure 1. (C) A phase diagram summarizes the expression phenotypes predicted by the Tat feedback model as basal transcription parameters (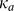 and 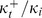) are varied over the observed experimental range of values while remaining model parameters are fixed. Drawn boundaries separate parameter combinations leading to distinct expression phenotypes. Model-predicted equilibration times (i.e., the time after which half of a Dim-initialized population crosses an intermediate expression threshold between Dim and Bright) are represented on a color scale, with longer times predicted for parameter combinations that specify Switching phenotypes. Parameter combinations used in (B) are marked with an asterisk.

We next developed a stochastic model of HIV transcription and amplification by the Tat positive feedback loop to aid our intuition concerning the underlying gene expression dynamics that may account for the observed variation in HIV expression phenotypes (Figure 2A). We previously built a model of basal LTR promoter-driven gene expression in the absence of Tat, which probabilistically described the processes of gene activation, transcription, and translation [22]. Our analysis suggested that basal transcription from the LTR occurs in short, infrequent bursts, and we found that the size of these transcriptional bursts strongly correlated with mean gene expression from different viral integration positions [22]. Here, we extended this basic model to include Tat expression from the LTR, and Tat positive feedback on transcription from the LTR, by assuming a Michaelis-Menten-like dependence of transcriptional burst size and burst frequency on Tat concentration (full model description included in Text S1). The assumption that Tat positive feedback enhances the frequency of transcriptional bursts from the LTR is consistent with observations that Tat interacts with transcription factors involved in gene activation [45], [46], and the assumption that Tat increases transcriptional burst size is based on observations that Tat enhances elongation by recruiting p-TEFb [39].

The model is specified by two basal transcription parameters, which set the average size and frequency of transcriptional bursts that occur in the absence of Tat, and three feedback parameters that describe transcriptional amplification in the presence of Tat. Two of these feedback parameters, which specify the average size and frequency of transcriptional bursts at saturating Tat concentrations (full transactivation), were set to give approximately a 100-fold increase in transcription rate at saturating Tat concentrations [40]. The third feedback parameter, which specifies the Tat concentration at half maximal binding, was set to approximately the top of the mid range of our bulk expression distributions (Figure 1B). The remaining model parameters (including degradation and translation rates) were set as in previous work [22]. The model, which was solved numerically for steady-state protein distributions, reproduced each of our major experimental expression phenotypes over different ranges of parameter values (Dim, Bright, and Switching (Figure 2B).

We qualitatively analyzed the relationship between transcriptional dynamics and expression phenotype in our model by generating a series of phase diagrams. These phase diagrams fix the Tat feedback parameters in our model as described above, and then systematically scan over basal transcription parameters, which are known to vary over genomic integrations [21], [22]. By applying our experimental criteria for Dim, Bright, and Switching phenotypes to our simulated distributions, we drew boundaries separating combinations of basal transcription parameters that lead to distinct expression phenotypes in our model (Figure 2C).

Interestingly, near the range of model parameters that generate Switching phenotypes, small changes in basal transcription that occur in the absence of Tat result in large changes in phenotype when amplified by Tat feedback (Figure 2B). Additionally, we found that Switching phenotypes exhibit delayed activation of gene expression. That is, if a simulated population of cells with model parameters corresponding to a Switching phenotype is initialized in the Dim state, a time-scale of one to many weeks is required for half of the population to cross a threshold of gene expression intermediate between Dim and Bright states (Figure 2B). This is in contrast to a Bright steady-state phenotype initialized in the Dim state, which will cross an intermediate expression threshold on a time scale of days (corresponding to the time scale of protein dilution in our cells). The delayed activation observed for the Switching phenotype is approximately the time scale over which an activated CD4+ T cell may transition to a memory state, and memory T cells are a primary reservoir of latent HIV infection *in vivo* [33], [34]. Thus, the delayed transcriptional activation exhibited by a Switching phenotype could substantially increase the opportunity for the memory state transition to occur in an infected T cell before viral production, and may therefore increase the probability of a latent infection.

The general relationship between Switching phenotypes and delayed activation is highlighted by superimposing a measure of distribution activation time on the phenotypic information in our phase diagrams (Figure 2C). Delayed activation results when transactivation depends on the probabilistic (infrequent) occurrence of multiple transcriptional bursts that are larger and/or more closely spaced than occur on average. In our model, such behavior occurs at intermediate values of basal transcriptional burst size and frequency, which are typically the same values that specify Switching phenotypes (additional discussion in Text S1). Our model thus supports the hypothesis that Switching phenotypes also exhibit delayed activation, which may underlie the establishment of latent HIV infections [20], [22], [43].

Finally, we note that Switching phenotypes also exhibit delayed deactivation of gene expression as compared to Dim clones when initiated in a Bright state. Although delayed deactivation is not relevant to the establishment of latent infections *in vivo* (due to the fact that viral replication would kill the host cell and block any possible memory state transition before deactivation could occur), it is possible to observe this behavior in our *in vitro* model. Thus, we hypothesized that probabilistic delays in both activation and deactivation can be used to select for Switching phenotypes in our *in vitro* system.

### Design of a dynamic forward genetic screen to select for promoter sequences that specify delayed activation and deactivation of viral gene expression

**Figure 3:**
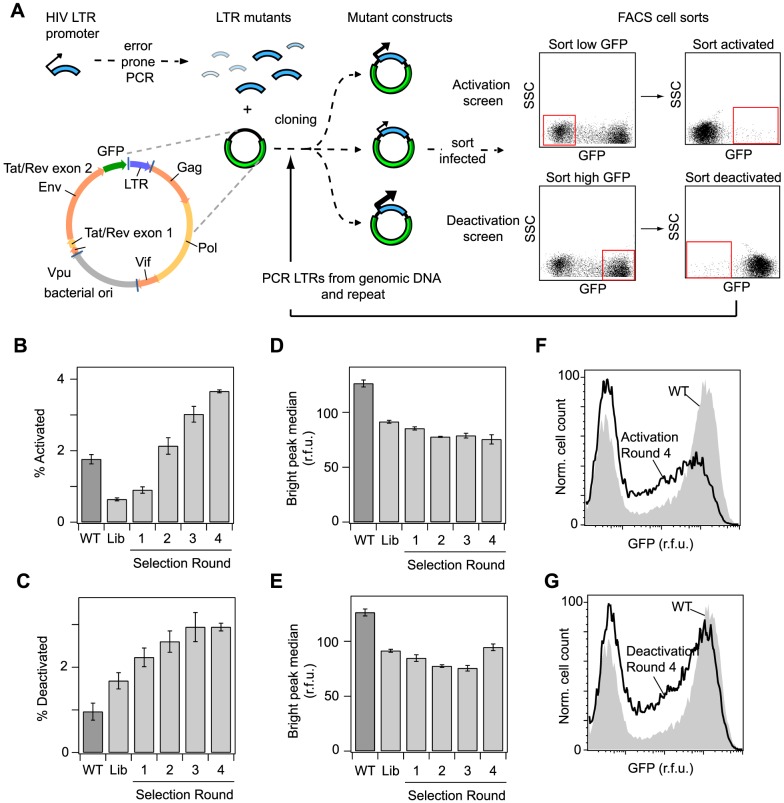
A dynamic forward genetic screen selects for LTR promoter sites that increase the frequency of delayed gene expression activation and deactivation. (A) Schematic of the genetic screen. (B–G) Jurkat cells were infected with the HIV lentiviral vector containing the WT promoter, the unselected library of promoters, or promoter libraries from each round of selection for delayed activation or deactivation. (B) Fraction of cells that showed delayed activation 5 days after sorting from the Dim gate. (C) Fraction of cells that showed delayed deactivation 5 days after sorting from the Bright gate. (D,E) Median GFP expression of the bright peak for promoter libraries selected from the (D) activation screen or (E) deactivation screen. All bar graphs are presented as the mean ± standard deviation of 3 replicates, and are representative of duplicate experiments. (F,G) Flow cytometry histograms comparing the WT initial bulk, multi-integration expression profile to the profile following four rounds of selection for (F) delayed activation or (G) delayed deactivation.

We exploited the delayed activation/deactivation of gene expression associated with Switching phenotypes to design a forward genetic screen to identify LTR promoter mutations that increase the prevalence of Switching phenotypes, and which could thus potentially influence the fraction of latent infections. We prepared a library of HIV-1 vectors in which the WT LTR promoter was subjected to random point mutations via error-prone PCR (Figure 3A) [47]. The ∼10^5^ member library had an average mutation rate of 0.6%, such that each position of the 634 base-pair promoter was mutated hundreds of times across the library. We packaged the library into our model vector, infected Jurkat cells, and isolated cell populations containing single viral integrations as described for the WT vector above. The resulting bulk population of singly infected cells, which was heterogeneous in both LTR sequence and viral integration position, was subjected to two alternate phenotypic screens. First, we implemented an ‘activation’ screen, in which infected cells with low GFP expression (low GFP gate) were isolated by FACS and allowed to grow for 5 days, at which point cells that had switched to high GFP expression (high GFP gate) were selected again by FACS. Second, a ‘deactivation’ screen reversed the order, selecting for high GFP expression first and low second (Figure 3A). We refer to the fraction of cells selected in these screens as the activating and deactivating fraction, respectively.

To confirm that our activation screen effectively selected for clones with a Switching phenotype, we applied the activation screen to the WT virus and randomly selected a sample of single cells from the activating fraction, which were then expanded to clonal populations for analysis. Remarkably, nearly 54% of these clones (22 out of 42) showed Switching phenotypes, as compared to only 8% from the original population and 19% from the mid-sorted population (Figure S1), confirming the effectiveness of the screen.

We thus implemented a larger scale analysis to identify viral promoter mutations that favor Switching phenotypes. Specifically, we performed multiple rounds of infection and FACS-based screening as described above to average the behavior of promoter sequences across different integration positions and thus identify genotypes that give rise to a higher fraction of Switching phenotypes across genomic contexts. After each round of infection, we recovered the viral LTRs from the genomic DNA of the selected populations (by PCR), re-cloned them into the sLTR vector, repackaged virus to produce a new library of selected promoters, and infected a new population of Jurkat cells (Figure 3A).

After four rounds of selection, the fraction of activating cells increased 6-fold compared to the original library (*p<0.001*, t-test on triplicate measurements) and 2-fold compared to the WT promoter (*p<0.01*; Figure 3B). The fraction of deactivating cells increased by a factor of 1.7 compared to the original library (*p<0.04*) and by a factor of 3 relative to WT (*p<0.002*; Figure 3C). Interestingly, the median GFP expression of the Tat-transactivated population (Bright peak in the bulk GFP histogram) was significantly lower for the unselected library than for WT, and it continued to decrease with each round of selection in both screens (Figure 3D–E). Importantly, the bulk gene expression distributions of the selected promoters also displayed an increased weight in the mid range of GFP expression (Figure 3F–G), which we had found to be enriched in integrations that demonstrate a Switching phenotype for the WT promoter. Altogether, these results indicate that our dynamic screens for activation and deactivation effectively selected for mutations that increased the fractions of activating and deactivating cells, which is a hallmark of the Switching phenotype.

### Genetic screens for delayed activation and deactivation of viral gene expression select for mutations in the core LTR promoter

**Figure 4:**
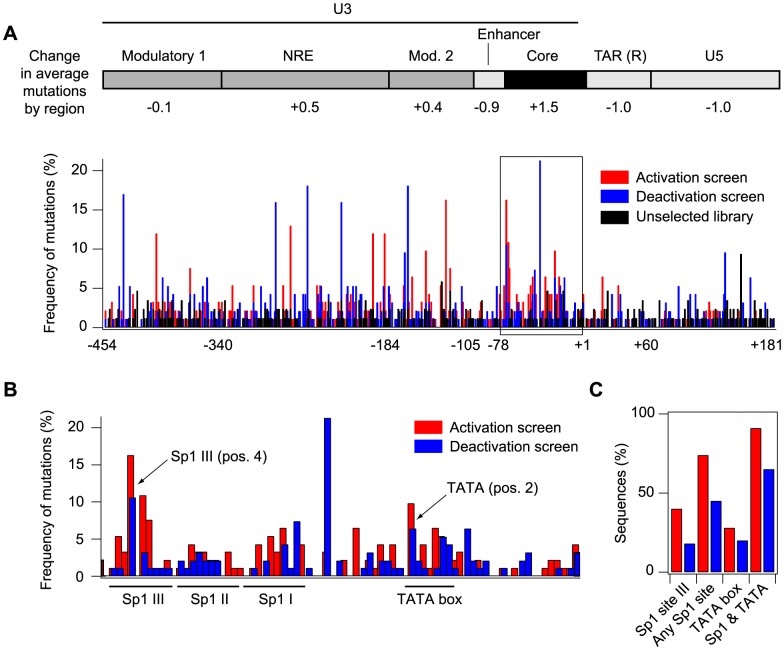
Genetic screen selects for mutations in the core LTR promoter. (A) Approximately 90 clones were sequenced per library of promoters. (Top) Sequenced clones from the activation and deactivation screens were combined and the distribution of mutations in functional regions of the LTR was compared to the distribution of mutations throughout the entire LTR. (Bottom) The frequency of mutations was plotted for each position of the LTR for the delayed activation screen (red), the delayed inactivation screen (blue), and the unselected library (black). (B) Frequency of mutations within the core promoter region for the delayed activation screen (red) and the delayed inactivation screen (blue). Arrows indicate the top two mutations that were selected in both screens. (C) Bar graph displaying the fraction of selected LTR sequences that have mutations in Sp1 site III or the TATA box for the activation screen (red) and the deactivation screen (blue).

To analyze the LTR promoter mutations that were enriched by the activation and deactivation screens, approximately 90 clones were sequenced from each selected library and compared to a control set of promoters from the unselected library. The average mutation frequency per position in the selected libraries was approximately 1.1% (as compared to 0.6% for the unselected library), but the distribution of mutation frequencies was long-tailed, with some positions mutated in as many as 20% of the promoters for a given screen (Figure 4A). We first analyzed how mutations were distributed across the LTR for the combined screens by comparing the mutation frequency for each regulatory region of the LTR with the average mutation frequency over the whole promoter [48] (Figure 4A). For both screens, mutations were most significantly enriched in the 78 base-pair core promoter region (*p*<0.0001, Chi-squared test), which includes transcription factor binding sites required for efficient promoter activation [48]. In contrast, mutation rates were not increased above those in the initial library in the enhancer region, the U5 region downstream of TAR, and in the TAR region itself, possibly reflecting the essential role of the TAR loop secondary structure to enable efficient gene expression [49]. The remaining regions displayed increased mutation frequencies that did not differ significantly from the average increase across the entire promoter for both selected libraries.

We next compared the mutation frequency at each position in the core promoter to the mutation frequency for the same base type in the unselected library (Figure 4B). We identified two positions in Sp1 site III, one position in Sp1 site II, and two positions in the TATA box with significant mutation rates in both screens (Table S2), with additional Sp1 and TATA positions significantly mutated in one of the two screens. The top hit was in Sp1 site III (position 4 of the 10 bp site, *p<0.0001*). Selection for this mutation is consistent with our previous results demonstrating that simultaneous mutation of positions 3 and 4 in Sp1 site III, which had been shown to eliminate binding of Sp1 [50], also increased delayed activation and deactivation in infected Jurkat cell populations [43]. The next strongest hit was in the TATA box (position 2 of the 8 bp site, *p = 0.0005*). The A to G mutation observed most frequently in our selected libraries has been previously shown to reduce the affinity of the TATA binding protein (TBP) for the TATA box [51]. Notably, mutations at positions 3 and 4 of the TATA box, which are considered critical for TBP binding and thus TATA function [51], [52], were not enriched in either screen.

Altogether, for the activation screen we found that 40% of the sampled sequences had mutations in Sp1 site III, and 25% had TATA mutations; for the deactivation screen, 20% had mutations in Sp1 site III, and 20% had TATA mutations (Figure 4C). All of these mutation frequencies were well above those for the same regions in the unselected library. Together, these results suggest the importance of Sp1 site III (and to a lesser extent the TATA box) in controlling stochastic gene expression and Switching fractions.

### Mutations in Sp1 site III and the TATA box increase the occurrence of Switching phenotypes

**Figure 5:**
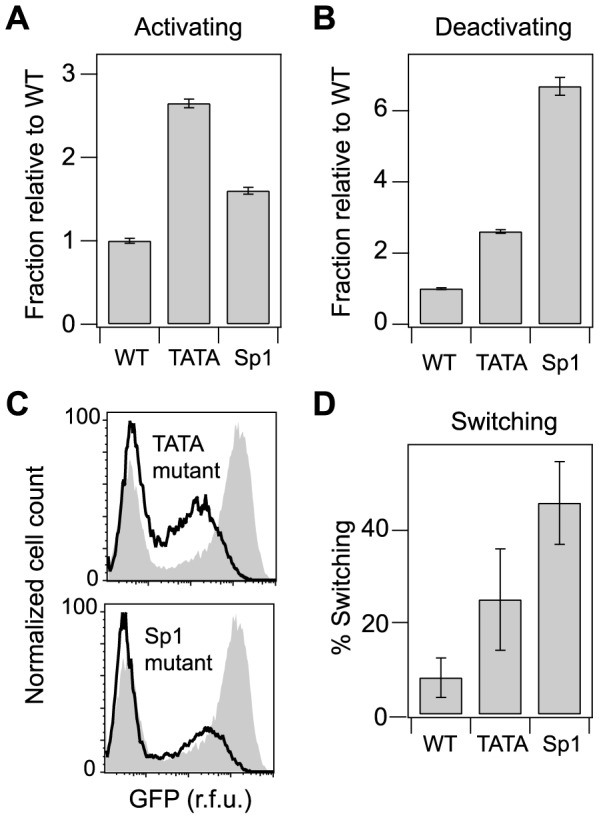
Selected mutations in Sp1 site III and the TATA box increase the Switching fraction. Jurkat cells were infected with the HIV lentiviral vector containing the WT promoter, with a single point mutation in Sp1 site III (position 4), or with a single point mutation in the TATA box (position 2). (A) Relative fraction of cells that activated 5 days after sorting from the Off gate. (B) Relative fraction of cells that deactivated 5 days after sorting from the Bright gate. (C) Flow cytometry histograms comparing the WT bulk-infection profile (gray) to the profile for TATAmutP2 (left) and Sp1mutIII (right). Note the reduced weight and position of the Bright (Tat-transactivated) peak and the increased weight of the mid region. (D) Switching fractions for WT and selected mutants. Approximately 80 clones were sorted from the mid region for each infected population, and the Switching fraction was estimated as described in the main text. Error bars indicate 95% CIs, estimated by a bootstrap method. Significant differences from WT (*p<0.01*) indicated by (*).

To directly analyze how the point mutations identified in our screen affect gene expression, we generated vectors with a single point mutation at position 4 of the Sp1 site III (Sp1 mutant) or at position 2 of the TATA box (TATA mutant) (Table S3), and infected Jurkat T cells as previously described. The TATA mutation increased both the activating and deactivating fractions of the infected population by approximately 2.5-fold relative to WT (*p<0.01*; Figure 5A–B), and the Sp1 mutation increased the activating fraction 1.5-fold (*p<0.03*; Figure 5A) and the deactivating fraction almost 7-fold relative to WT (Figure 5B, *p<0.001*). Both point mutations also significantly decreased Tat-mediated gene expression and increased expression in the mid-range of fluorescence (Figure 5C), mirroring the bulk expression phenotype of the full library after selection, and consistent with previous studies [43], [53], [54].

We next quantified Switching fractions for both mutants by sorting approximately 80 single-integration clones from the mid-range of GFP in the bulk populations as previously described for the WT virus (Figure 1). The Switching fractions increased from 8% for the WT virus to 25% for the TATA mutant and 46% for the Sp1 mutant (Figure 5D). These results confirm that increased activation and deactivation in the bulk infection for these mutants reflect an increased frequency of single-integration clonal Switching phenotypes (*p<0.01*, bootstrap method).

### Selected mutations in Sp1 site III result in small but significant differences in basal gene expression dynamics across integration positions

We next considered how promoter mutations might alter transcriptional dynamics to increase the fraction of infections that generate Switching phenotypes. For this analysis, we chose to focus on the Sp1 point mutation, because this point mutation exists in naturally occurring HIV isolates, while the TATA mutation was not found (as determined by searching the Los Alamos HIV sequence database, http://www.hiv.lanl.gov). Furthermore, our previous work also demonstrated a role for Sp1 site III in regulating Switching phenotypes [43].

**Figure 6:**
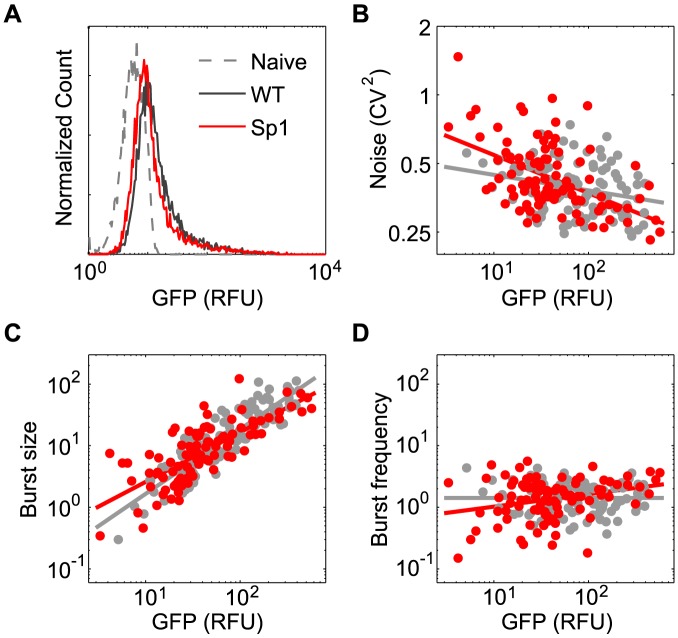
Selected mutations result in small but significant differences in basal gene expression. (A) Flow cytometry bulk-infection histograms for Jurkat cell populations. Each cell contains a single (different) integration of the Tat-null vector (sLTR-GFP-TatKO) with a WT LTR promoter (black), or an LTR with an Sp1 site III mutation (red). Uninfected Jurkat histogram is displayed for reference (gray). (B–D) Distribution noise (defined as CV^2^) versus mean GFP for Sp1 mutant clones sorted and expanded from the bulk populations in (A). (C–D) Clonal histograms were fit with the stochastic gene-expression model in the absence of feedback (Figure 2A), and best-fit parameters were calculated for (C) transcriptional burst size and (D) transcriptional burst frequency. Each point in B–D represents a single-integration clone from a WT (gray) or Sp1 mutant (red) infection.

Our earlier work demonstrated that basal transcription (i.e. in the absence of Tat) varies significantly with integration position of the LTR [22]. Therefore, we hypothesized that Sp1 may modulate phenotypic distributions by directly affecting basal transcription. To test this hypothesis, we introduced stop codons into the first Tat exon of the lentiviral vector backbones of the WT and the Sp1 mutant promoter and infected Jurkats as described above (Figure 6A). Bulk-infection expression distributions for both Tat-null vectors demonstrated substantial overlap with autofluorescence controls, but with a strong right skew towards higher fluorescence. Notably, a small but significant decrease in mean GFP expression was observed for the Sp1 mutant promoter compared to WT (*p<0.05*), consistent with previous studies [53], [54]. Additionally, clonal cell populations expanded from each bulk population had monomodal, wide, right-skewed distributions (Figure S3) and displayed high levels of noise across clonal expression means (Figure 6B), consistent with previous results for the WT LTR promoter [22].

To infer the underlying transcriptional dynamics of our Tat-null clones, we systematically fit their GFP distributions using our model (Figure 2A with transactivation removed), following our earlier analysis of WT basal expression dynamics [22]. The sets of clonal WT and Sp1 distributions were all best accounted for by a bursting dynamic, whereby short infrequent transcriptional bursts generate large basal expression heterogeneities (see Text S1 and [22] for further discussion). The basal transcription dynamics for each clonal population were fully quantified by a best-fit basal transcriptional burst size and burst frequency. Transcriptional burst sizes were found to vary from a few to tens of transcripts, and to be strongly positively correlated with mean expression level across different integration positions for both the mutant and for the WT vector (Figure 6C). In contrast, typical transcriptional burst frequencies were on the order of a few events per cell division time, and demonstrated little correlation with mean gene expression levels over integration positions (Figure 6D). These findings are consistent with our earlier analysis of the WT promoter [22].

Although the Sp1 mutant and WT promoters share the same qualitative basal expression dynamics, regression analysis revealed that the Sp1 mutant demonstrated an increased positive correlation between basal burst frequency and clonal expression mean, with burst frequencies decreased for Dim clones (Figure 6D; *p* = 0.04). Thus, the selected Sp1 mutation does not change the qualitative bursting mode of transcription from the HIV LTR, but it does appear to modestly alter how the dynamics vary quantitatively across integration positions.

### Altered basal gene expression dynamics for the Sp1 mutation may contribute to Switching-phenotype enrichment

**Figure 7:**
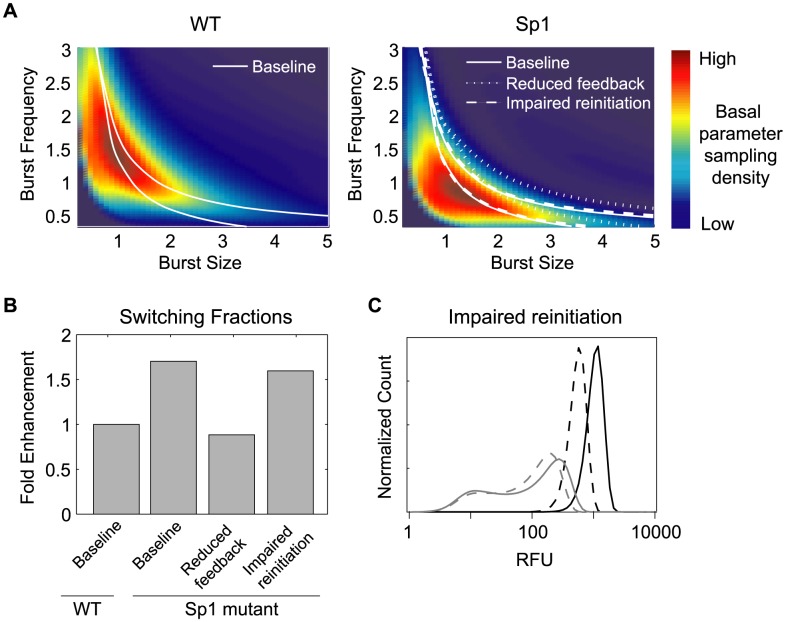
Computational models exploring Switching fraction modulation by the Sp1 mutation. (A) Model phase diagrams varying basal transcriptional parameters at fixed values of Tat feedback parameters. Drawn boundaries separate parameter combinations leading to distinct phenotypes (as in Figure 2C). Superimposed color map estimates the probability density with which the virus samples basal transcription parameters over genomic integrations for the WT promoter (left) and Sp1 mutant promoter (right). Tat feedback parameters that result in a WT Switching-fraction estimate of 12% specify the solid phenotypic boundaries (base). Decreasing the fold-amplification of Tat feedback (reduced feedback, short dashed lines) shifts phenotypic boundaries to the right, while impaired reinitiation (long dashed lines) has little effect on phenotypic boundaries. (B) Estimated Switching fractions for the sets of Tat feedback parameters used in (A), normalized by the predicted WT Switching fraction for the base set of parameters (solid line). (C) Sample Switching (grey) and Bright (black) distributions for the base set of Tat feedback parameters (solid) and for impaired reinitiation parameters (dashed). The degree of transcriptional reinitiation impairment was chosen to produce a comparable shift in Bright phenotype as the parameters for reduced feedback (A–B). The model extension to include transcriptional reinitiation was implemented by a simple rescaling of model parameters according to: 
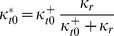
 (rescaled basal transcription rate); 
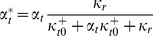
 (rescaled amplification factor for transactivated transcription rate); 
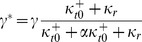
 (rescaled feedback saturation parameter). Details may be found in Text S1.

We returned to our model to explore if the small changes in basal transcriptional dynamics quantified experimentally with our Tat-null vector could contribute significantly to the increased Switching fraction observed for the Sp1 mutant in the presence of Tat (Figure 5D). The phase diagrams developed for the WT promoter (Figure 2C) specify the predicted expression phenotype for every combination of basal transcriptional burst size and burst frequency parameters for fixed Tat feedback. Thus, model phase diagrams can be used to predict the Switching fraction that would result from a given probability density with which the virus samples basal transcriptional parameters through its sampling genomic locations via infection and integration, under the assumption of fixed Tat feedback. We used our experimental data to estimate the probability density with which the WT and Sp1 mutant promoters sampled combinations of basal transcription parameters (see Text S1 for details), and then calculated model-predicted Switching fractions by integrating this sampling density over the Switching region of the phase diagram (Figure 7A).

We found that the changes in basal transcriptional dynamics observed for the Sp1 mutant – particularly the increased sampling of lower transcriptional burst frequencies, which specify noisier basal transcription – indeed resulted in higher model-predicted Switching fractions compared to WT for all sets of feedback parameters analyzed. In particular, for a set of feedback parameters that specify a model-predicted Switching fraction of 12% for the WT basal parameter sampling density, the model predicted a Switching fraction of 22% for the Sp1 mutant sampling density (Figure 7B). Thus, we conclude that changes in Sp1 basal transcription dynamics can result in a substantial increase in the fraction of genomic integrations that lead to a Switching phenotype in the presence of Tat feedback.

### Attenuated Tat positive feedback resulting from impaired transcriptional reinitiation can preserve increased Switching fractions for the Sp1 mutant

In addition to altering basal expression, mutations in Sp1 site III weaken Tat positive feedback, as demonstrated in our experiments (Figure 5C) and in previous work [53]; however our model had not yet accounted for this observation. We therefore explored if weakening Tat positive feedback in the model would maintain the predicted Switching fraction enrichment that arises from altered basal transcription, or even enhance it to more fully account for the nearly 6-fold enrichment observed in our experiments. In contrast to these expectations, we found that decreasing Tat-driven fold-amplification of basal transcription in the model typically decreased predicted Switching fractions (Figure 7B), a result which can be explained by our model phase diagrams (Figure 7A). Notably, weakening feedback shifts phenotypic boundaries to the right (towards larger basal transcriptional burst sizes), transforming Bright integrations to Switching, and Switching to Dim. The resulting Switching region typically enclosed a smaller fraction of the viral basal parameter sampling density, which is highly right skewed and heavily weighted at lower basal transcriptional burst sizes. Thus, our analysis suggests that the Sp1 site mutation specifies a more complex perturbation of the Tat positive feedback loop that differentially affects Bright and Dim integrations, rather than one that uniformly attenuates expression amplification over genomic integrations.

A biological mechanism by which the Sp1 site mutation could differentially affect Bright and Dim integrations is by impairing transcriptional reinitiation. In the bursting model of transcription, each gene activation event can drive multiple cycles of transcription, requiring multiple rounds of RNAPII binding and transcription-complex formation (i.e. reinitiation). In the absence of Tat, the rate-limiting step in HIV-LTR transcription is RNAPII stalling at the TAR hairpin that forms after transcriptional initiation [38]. Therefore, moderate impairment of the reinitiation rate via mutation would be masked during basal transcription, or for integrations that inefficiently activate Tat feedback. However, at higher concentrations of Tat, when the TAR-loop stall is no longer rate limiting, impaired reinitiation would significantly attenuate full Tat transactivation, and the effect would be more pronounced for Brighter genomic integrations. Because Sp1 and p-TEFb interact *in vivo* to activate HIV transcription [46], [55], [56], a mutation in the Sp1 site could plausibly alter transcriptional reinitiation if it disrupted recruitment of p-TEFb.

To investigate this possibility, we extended our model to include a ‘reinitiation’ step between each transcript production event (rescaled model parameters included in Figure 7 legend and full model description and equations included in Text S1). The effective transcript production rate in this extended model depends on both an elongation rate, which varies over genomic integrations, and a reinitiation rate, which is fixed (but may be altered through mutation). The elongation rate specifies the variation of the basal and transactivated transcription rates over genomic integrations, while the reinitiation rate specifies the maximal value at which the transcription rate saturates as a function of elongation rate. In this extended model, we found that a moderate decrease in the transcriptional reinitiation rate had little effect on the phenotypic boundaries of our phase diagrams (Figure 7B), but significantly weakened Tat-transactivated expression from Bright integrations (Figure 7C), consistent with our experimental observations (Figure 5C). As a result, predicted Switching fractions were preserved, though they were not further enhanced to the level observed experimentally. Thus, moderate impairment of transcriptional reinitiation could account for the observed attenuation in Tat-mediated gene expression (Figure 7B), while preserving (but not increasing) the Switching fraction enhancement that was predicted for the observed changes in Sp1 mutant sampling of basal transcription parameters.

## Discussion

Amplification of HIV gene expression noise via Tat positive feedback results in a wide range of noise-driven phenotypes that vary across the diverse host genomic environments sampled during HIV infection. Here, using an *in vitro* cell-based HIV model system and a novel forward genetic screen, we identified LTR promoter mutations that increase the frequency of the Switching phenotype, a model for latent viral infections. Two key features of our screen are 1) its dynamic nature, which selects for stochastic phenotypes that ‘switch’ between quiescent and highly expressing states; and 2) integration randomization, which applies selective pressure on mutations affecting the fraction of integrations that specify Switching phenotypes rather than on the integration positions themselves. These features reflect the time-varying selective pressure that is likely applied by a dynamic immune system and therapy schedule, and the integration randomization that occurs when a viral lineage is propagated by new infections *in vivo*. Our forward genetics approach enabled the systematic identification of promoter elements that affect the Switching fraction, and complements prior reverse genetics approaches that analyzed how specific mutations affect gene expression and phenotype [43], [53], [57]. The screen identified strongly selected mutations in Sp1 and TATA transcription factor binding sites within the core transcriptional regulatory region of the HIV LTR, and we confirmed that these mutations led to higher frequencies of Switching phenotypes across integration positions.

### Integrating models and measurements to identify biological mechanisms underlying experimental observations

Our study was enabled by the development of a computational model that described how promoter-driven expression fluctuations are propagated via Tat positive feedback to generate the wide range of expression phenotypes in our system. We used this model to investigate features of Tat feedback that generate stochastic phenotypes, to formulate hypotheses concerning the mechanisms by which these features may be varied through mutation, and to study the implications and consistency of these hypotheses with our experimental data.

The Tat transactivation circuit – an essential and conserved feature of the HIV virus across clades – is characterized in our model by positive feedback loops that enhance both the size and frequency of transcriptional bursts. HIV gene expression phenotypes range from Dim to Bright as the kinetic parameters of the circuit are varied, with intermediate parameter values generating the stochastic Switching phenotypes that our screen was designed to select. These Switching phenotypes, which we have suggested may serve as a model for latent infection [20], [22], [43], are characterized by Tat-amplified transcriptional fluctuations that drive stochastic switching between quiescent and highly expressing states (Figure 2). Importantly, all of the transcriptional and regulatory processes described in our model – and their underlying kinetic parameters – can be modulated by genomic environment. Thus, a viral sampling of genomic environments that range from repressive to permissive can tune the steady-state behavior of Tat positive feedback circuit to generate a distribution of expression phenotypes that span from Dim to Bright, with intermediate integrations generating Switching phenotypes [41]. In this way, the possibility of stochastically-generated latent phenotypes at a subset of viral integrations may be an intrinsic feature of the Tat circuit and its sampling of host-cell genomic environments, and the virus may tune the fraction of integrations that specify this phenotype through mutation.

Guided by our model analysis both here and in previous work [22], we hypothesized that the Sp1 mutation may alter the prevalence of Switching phenotypes by modulating basal transcription dynamics. Although the underlying basal bursting dynamic of the WT promoter was essentially preserved in the Sp1 mutant (Figure 6), we were able to detect modest quantitative differences in the sampling of basal expression dynamics over integration positions. Our computational analysis confirmed that these small differences in basal expression for the Sp1 mutant could be amplified in the presence of Tat feedback to produce substantial increases in the Switching fraction (Figure 7B).

The selected Sp1 mutant also demonstrated weaker Tat-transactivated expression, and we further used our model to investigate how this could affect the Switching fraction. Our model analysis demonstrated that weakening Tat feedback proportionately for all integrations would decrease, rather than increase, Switching fractions (Figure 7B). Thus, accounting for an increased Switching fraction in the presence of weaker Tat feedback required a mechanism by which the selected mutation could differentially affect basal and transactivated expression, which we suggested could be accomplished through impaired transcriptional reinitiation. A revised computational model that included impaired transcriptional reinitiation could thus account qualitatively for both trends observed experimentally for the Sp1 mutant: an enhanced Switching fraction accompanied by attenuated Tat-transactivated expression (Figure 7B–C). However, we note that our model still does not quantitatively account for the full increase in Switching fraction observed experimentally for the Sp1 mutant (Figure 5D). A complete explanation might thus require identification of additional mechanisms that differentially affect Tat transactivation across genomic integrations and a more detailed characterization of how the selected mutations perturb the transcription parameters sampled by the virus over genomic integrations.

### Mechanisms by which Sp1 and TBP may control HIV expression phenotypes

Multiple studies have demonstrated that mutations in the Sp1 sites of the HIV LTR can significantly reduce HIV Tat-mediated transactivation, while minimally affecting basal expression (for those cases in which it was measured) [53], [55], [58], [59]. Although the detailed mechanisms by which Sp1 regulates HIV expression remain unknown, there is evidence that Sp1 recruits P-TEFb *in vivo* to release the stalled RNAPII from the promoter proximal region and activate transcriptional elongation of HIV [46], [55], [56]. To our knowledge, a role for Sp1 in transcriptional reinitiation has not been directly tested. However, if Sp1 participates in recruitment of P-TEFb, then lower affinity Sp1 binding (caused by promoter mutation) may destabilize the P-TEFb complex in the promoter active state and thus lower the rate of transcriptional reinitiation (κ_r_ in our model).

Interestingly, TATA mutations in the HIV LTR also substantially reduce Tat-mediated transactivation without affecting mean basal expression from the HIV LTR [53], [54], [60], [61], similar to observations by others and us for Sp1 mutation. Although we did not explore the mechanisms underlying mutation of the TATA box, an increase in the half-time of transcriptional reinitiation (1/κ_r_ in our model) has been measured directly for a mutation at site 2 of the TATA box [62]. Furthermore, TATA box mutations that decreased reinitiation also correlated with decreased stability of the TBP:TFIIA (general transcription factor) complex on the DNA, suggesting that retention of general transcription factors at the promoter is a primary determinant of the reinitiation rate [63]. Our results motivate a future experimental study that directly measures if reduced transcriptional reinitiation provides a mechanistic explanation for the differential effect of Sp1 and TATA box mutations on basal and Tat-transactivated HIV transcription, as observed here and in many previous studies [53], [55], [58], [59].

### Implications for our understanding of HIV latency

*In vivo*, infected CD4+ T cells that have transitioned to a memory state form a primary reservoir of latent infection [33], [34]. However, HIV does not efficiently establish infection in resting memory CD4+ T cells [64], [65], and activated CD4+ T cells typically die within days after infection [30]. Therefore, we hypothesize that transcriptional delays, such as those associated the Switching phenotype in our *in vitro* system and that occur on a similar time scale to the memory-state transition, could delay viral production and thus increase the time window during which the memory-state transition could occur post-infection. Thus, viral mutations such as the Sp1 and TATA mutations identified in our study, which result in an increased fraction of viral integrations demonstrating transcriptional delays, could lead to an increase in the fraction of memory T cells that harbor a latent infection.

If this *in vitro* model of latency has *in vivo* implications, then our results suggest that there may be enrichment for viruses with an Sp1 and/or TATA box mutation in the latent reservoir. Although we are unaware of any direct evidence of enrichment for either Sp1 or TATA box mutations in the latent pool, there is evidence that viruses with an Sp1 site III mutation are enriched during the course of disease progression [66] and that viruses with impaired Tat activity are enriched in latent reservoirs [67]. These studies are suggestive that some viral mutations, particularly ones affecting Tat transactivation as demonstrated in our study, may create favorable conditions for establishing latent infections. Interestingly, these studies suggested that lower transcriptional activity may underlie the propensity of these viruses to establish a latent infection, but our results suggest it is instead the increased probability for transcriptional delay that potentiates latent infection. A related and testable hypothesis is that the three HIV subtypes (D, F and H) with mutations in Sp1 site III may demonstrate an increased propensity for latency and thus give rise to larger latent reservoirs relative to subtype B infection. To our knowledge, there is no study that has examined the relative sizes of the latent viral reservoirs for different HIV subtypes, and therefore this may be an important translational study that is motivated by our work.

In conclusion, our study provides an integrated experimental and computational framework for identifying genetic sequences that alter the distribution of stochastic expression phenotypes over genomic locations and for characterizing their mechanisms of regulation. Our results also may yield further insights into the mechanisms by which HIV sequence evolution can alter the propensity for latent infections.

## Methods

### Cell culture

HEK293T cells (ATCC) were cultured in IMDM (Mediatech) and Jurkat clone E6 cells (ATCC) were cultured in RPMI (Mediatech). All media was supplemented with 10% FBS (Gibco) and 100 U/ml penicillin+100 mg/ml streptomycin (Gibco). Jurkat cell concentrations were maintained between 2×10^5^ and 2×10^6^ cells/ml in 5% CO_2_ at 37°C.

### Viral cloning and packaging

We modified a full-length single-LTR packaging platform described previously in which HIV Nef was replaced with GFP [44]. Multiple stop codons were introduced into all viral proteins except Tat (psLTR-Tat-GFP; Table S4) using Quickchange site-directed mutagenesis (Stratagene). To generate Tat-null sequences, additional stop codons were introduced into the first exon of Tat (psLTR-TatKO-GFP). The LTR promoter library was amplified in an error-prone PCR reaction described previously [47] using Taq DNA polymerase with 2% MnCl_2_. The resulting promoter library was cloned into the psLTR-Tat-GFP by restriction digest with PmeI and KasI. Following each round of selection, the genomic DNA from the selected cells was isolated using a QiaAMP DNA Micro Kit (Qiagen) and the LTR promoters of the integrated proviruses were amplified with primers that retained the PmeI and KasI restriction digest sites for cloning. Single point mutations in the LTR were introduced with Quickchange site-directed mutagenesis (see Table S3 for sequences) and each mutant LTR was sequenced and subcloned back into the parental plasmid to avoid unintended mutations. All psLTR-Tat-GFP and psLTR-TatKO-GFP plasmids were packaged and harvested in HEK 293T cells with helper plasmids (pcDNA3 IVS VSV-G, pMDLg/pRRE, pRSV Rev, and pCLPIT-tat mCherry) as previously described [20], [68]. Harvested lentivirus was concentrated by ultracentrifugation to yield between 10^7^ and 10^8^ infectious units/ml. To titer, Jurkat cells were infected with a range of vector concentrations and six days post infection, gene expression of infected cells was transactivated by stimulation with 20 ng/ml PMA (Sigma) and 400 nM TSA (Sigma). After stimulation for 18–24 hours, GFP expression was measured by flow cytometry, and titering curves were constructed by determining the percentages of cells that exhibited GFP fluorescence greater than background levels.

### Library selections and analysis

Jurkat cells were infected with the sLTR-Tat-GFP virus at an MOI of ≤0.1 and cultured for 7–10 days. Cells were stimulated with 20 ng/ml TNF-α (Peprotech) for 18–24 hours and GFP+ cells were sorted on a MoFlo Cell Sorter (Cytomation). Sorted cells were cultured for 10 days. For the activation screen, cells were sorted from the off peak (bottom third of the full range of GFP expression), cultured for 5 days, and then selected as positive for enrichment if the cells activated above the mid-point of the expression range. For the inactivation screen, cells were sorted from the bright peak (top third of the full range of GFP expression), cultured for 5 days, and then selected as positive for enrichment if the cells inactivated below the mid-point of the expression range. Flow cytometry data analysis was performed with FlowJo (Tree Star, Inc.).

### Selection of infected clones

For LTR-Tat-GFP infections, single cells were selected from the region of interest (bottom third of the expression distribution for off cells, mid third of the expression distribution for bimodal cells, and top third of the expression distribution for bright cells). For the LTR-Tat-null vector, single cells were selected from either the top 10% or 18% of the GFP expression distribution and sorted into each well of a 96-well plate on a MoFlo Cell Sorter (Cytomation). Clonal cells were cultured for 2–3 weeks and then analyzed on an FC500 flow cytometer (Becton Dickenson).

### Clonal phenotypic determination and Switching-fraction estimation

Fluorescence histograms for single-integration clonal sLTR-Tat-GFP infections were labeled as Switching if they exceeded specified cut-offs in any of the following distribution features: inter-quartile range, cube root of 3^rd^ central moment, peak separation and dip for bimodal distributions, and the product of distribution weight in approximately the lower third and upper half of our cytometer log fluorescence range. Feature cut-offs were specified by visualizing the full set of clonal distributions using k-means clustering based on 8 distribution features normalized by inter-quartile range (those mentioned, and mean log fluorescence, distribution weight in the lower 3^rd^ of the bulk fluorescence range, and distribution 4^th^ central moment) using 20 clusters and a Euclidean distance, implemented in Matlab (The Mathworks). Sorting clusters separately by each feature centroid allowed identification by eye of features and cut-off values beyond which all distributions could be labeled as Switching. This approach extended our by-eye intuition from distributions whose phenotype could be unambiguously scored by eye to those whose phenotype was ambiguous (see Text S1 for further details). Key results, such as Switching fraction enrichment for our analyzed mutants, were robust to variation of feature cut-off values.

Switching fractions, over the full set of genomic integrations, were estimated from mid-sorted sub-samples, via an application of Bayes theorem:
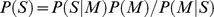
where *S* is the event that an infected cell contains a Switching integration and *M* is the event that the cellular fluorescence is in the range of the sorting gate (i.e. mid range). The conditional probability, 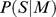, was estimated as the fraction of clones from a given mid sort that were labeled as Switching (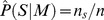 where 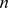 is the total number of clones analyzed from the mid sort and 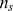 is the number that were labeled as Switching). The probability that a cell expresses fluorescence in the range of the sort, 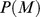, was estimated by the distribution weight of the bulk multi-integration population in the sort range. 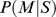, the distribution weight in the sort region for the full population of Switching integrations, was estimated from our mid-sorted set of Switching clones as:
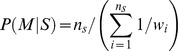
where the 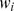 are individual distribution weights of the mid-sorted Switching clones in the sort region. Uncertainties in Switching-fraction estimation were calculated based on a bootstrap approach [69]. Further details are provided in Text S1.

### Computational modeling and distribution fitting

Our model of the transactivation circuit considers each reaction as a Markov process, proceeding with fixed probability per unit time (full model details in Text S1). For any fixed set of parameter values, the model was solved to obtain predicted steady-state protein distributions across a clonal population of cells by approximating and numerically integrating the master equation for the system [70] in time until a stationary distribution was achieved. Protein numbers were convert to cytometer RFU by scaling, and distributions were convolved with a measured autofluorescence profile for comparison with experimental distributions, following [22].

Tat-null distributions were fit to the transactivation model with feedback from Tat removed based on the first 6 distribution moments (see Supplemental text for further details). Transcriptional bursting was assumed, so that transcriptional burst size (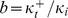) and burst frequency (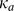) were the only model fit parameters, with the remaining model parameters calibrated following [22]. The assumption of transcriptional bursting was checked by systematically varying the active-state duration (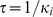) and refitting the model at each value. Consistent with [22], the best fits were always found in the transcriptional bursting regime (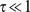). All analysis was done using in-house code written in Matlab (The Mathworks).

### Statistical analysis

Statistical significance of differences between means in triplicate experiments was assessed using a 2-sided t-test. Pearson Chi-squared statistics were calculated for the appropriate contingency tables to assess differences in mutation rates between libraries marginally and by regulatory region, and at individual positions along the promoter, after controlling for base type in the WT (parent) sequence. All quoted raw p-values for post-hoc analysis remain significant at the 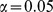 level for Type I error after Bonferroni correction, and corresponding global tests were always significant at least at this level. Equality of regression coefficients was assessed by partial F-test, and differences between individual regression parameters were assessed by t-test in post-hoc analysis. Confidence intervals for experimental Switching fraction estimates, and p-values for their differences, were estimated using a bootstrap procedure. Contingency table analysis was conducted using SAS/STAT software version 9.1 for Windows, Copyright 2012 SAS Institute Inc. All other computational analysis was performed using Matlab (The Mathworks).

## Supporting Information

Figure S1 Enrichment for wide/bimodal (i.e., “Switching”) phenotype. WT clones were isolated by FACS using three different sorting methods: 1) cells sorted from the entire infected population; 2) cells sorted from the mid-GFP region of the infected population; and 3) cells sorted from the activating fraction after one round of selection. Switching fraction was estimated as described in Text S2. Error bars indicate 95% CIs, estimated by a bootstrap method.
(PDF)

Figure S2 Clonal-distribution clustering for phenotypic determination. A) Frequency histograms of distribution features over the full set of clones analyzed for the Tat feedback vector in this study, with dashed lines marking cut-off values that were used for phenotypic specification. B) Full set of clustered, relative-frequency, clonal expression distributions, with distributions phenotypically labeled as Dim (blue), Bright (green), or Switching (red). Clusters are ordered by centroid value for the IQR feature. C) Heat-map representation of clustered clonal expression histograms, with clusters ordered as in B, with distributions ordered within clusters by IQR. To better visualize wide distributions, a count of 1 was added to each histogram bin, and the log count was represented in a color map, normalized between the minimum and maximum count for each clonal histogram.
(PDF)

Figure S3 Log-binned histograms of clones infected with the WT Tat-null vector and the Sp1 mutant Tat-null vector. Clonal distributions were monomodal and wide with highly skewed distributions, which becomes apparent upon transformation to a real fluorescence axis.
(PDF)

Table S1 Features used in cluster-based analysis of clonal populations
(PDF)

Table S2 Positions in the LTR with significant mutation rates following selection.
(PDF)

Table S3 Point mutations introduced into LTR promoters used in our experimental studies.
(PDF)

Table S4 Sequences of HIV genes up to and including stop codons used in the sLTR-Tat-GFP vector.
(DOCX)

Text S1 Supporting computational methods. This text contains explanatory notes on feature-based clustering, estimates of Switching fraction, details of the Tat feedback model, including model equations and parameters.
(PDF)
