# Supplementary material for: Genetic Selection for Context-Dependent Stochastic Phenotypes: Sp1 and TATA Mutations Increase Phenotypic Noise in HIV-1 Gene Expression
Source: PLoS Comput Biol. 2013 Jul 11;9(7):e1003135. doi: 10.1371/journal.pcbi.1003135 (PMC3708878; doi:10.1371/journal.pcbi.1003135)
Supplement: Figure S1 — Enrichment for wide/bimodal (i.e., “Switching”) phenotype. WT clones were isolated by FACS using three different sorting methods: 1) cells sorted from the entire infected population; 2) cells sorted from the mid-GFP region of the infected population; and 3) cells sorted from the activating fraction after one round of selection. Switching fraction was estimated as described in Text S2. Error bars indicate 95% CIs, estimated by a bootstrap method. (PDF) [file pcbi.1003135.s001.pdf]

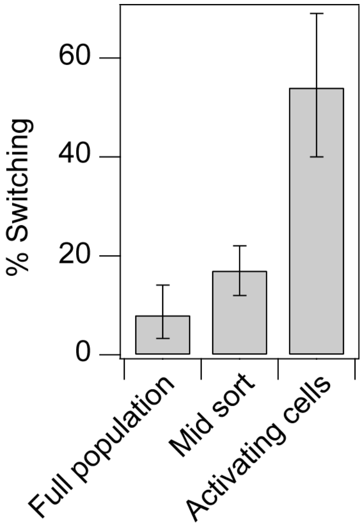

Figure S1. Enrichment for wide/bimodal (i.e., "Switching") phenotype. WT clones were isolated by FACS using three different sorting methods: 1) cells sorted from the entire infected population; 2) cells sorted from the mid-GFP region of the infected population; and 3) cells sorted from the activating fraction after one round of selection. Switching fraction was estimated as described in Text S2. Error bars indicate 95% CIs, estimated by a bootstrap method.
