# Supplementary material for: Genetic Selection for Context-Dependent Stochastic Phenotypes: Sp1 and TATA Mutations Increase Phenotypic Noise in HIV-1 Gene Expression
Source: PLoS Comput Biol. 2013 Jul 11;9(7):e1003135. doi: 10.1371/journal.pcbi.1003135 (PMC3708878; doi:10.1371/journal.pcbi.1003135)
Supplement: Figure S2 — Clonal-distribution clustering for phenotypic determination. A) Frequency histograms of distribution features over the full set of clones analyzed for the Tat feedback vector in this study, with dashed lines marking cut-off values that were used for phenotypic specification. B) Full set of clustered, relative-frequency, clonal expression distributions, with distributions phenotypically labeled as Dim (blue), Bright (green), or Switching (red). Clusters are ordered by centroid value for the IQR feature. C) Heat-map representation of clustered clonal expression histograms, with clusters ordered as in B, with distributions ordered within clusters by IQR. To better visualize wide distributions, a count of 1 was added to each histogram bin, and the log count was represented in a color map, normalized between the minimum and maximum count for each clonal histogram. (PDF) [file pcbi.1003135.s002.pdf]

A

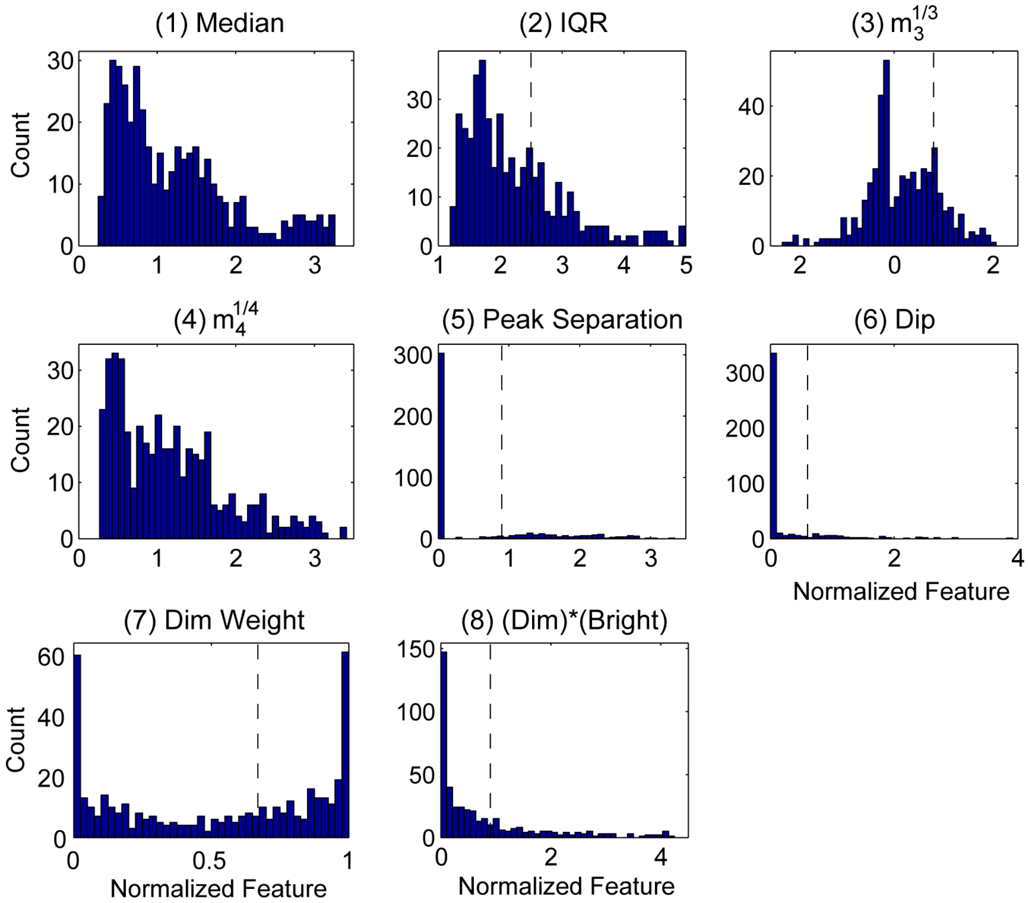

Figure S2. Clonal-distribution clustering for phenotypic determination. A) Frequency histograms of distribution features over the full set of clones analyzed for the Tat feedback vector in this study, with dashed lines marking cut-off values that were used for phenotypic specification. (Continued next page)

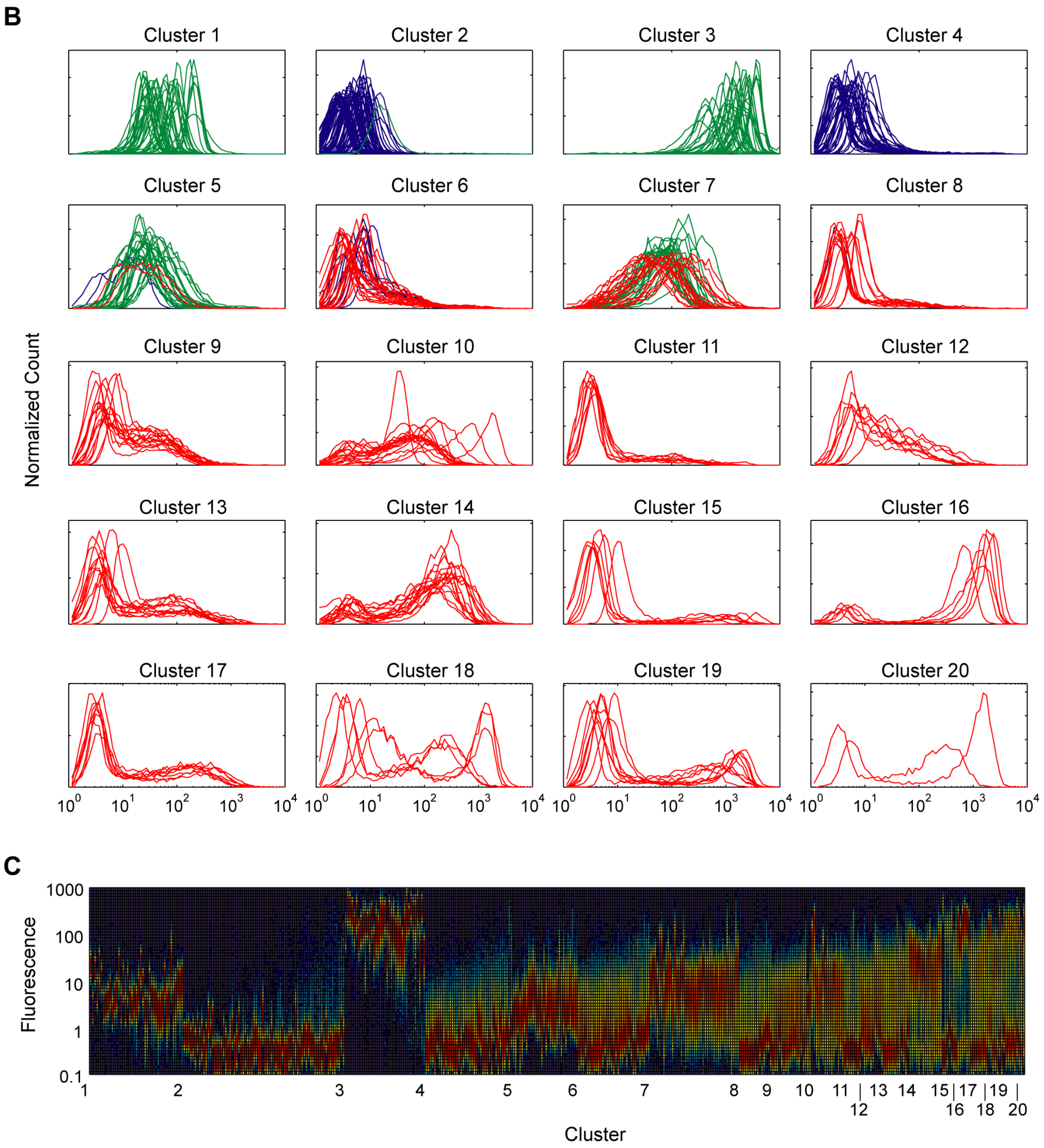

Figure S2 (continued). B) Full set of clustered, relative-frequency, clonal expression distributions, with distributions phenotypically labeled as Dim (blue), Bright (green), or Switching (red). Clusters are ordered by centroid value for the IQR feature. C) Heat-map representation of clustered clonal expression histograms, with clusters ordered as in B, with distributions ordered within clusters by IQR. To better visualize wide distributions, a count of 1 was added to each histogram bin, and the log count was represented in a color map, normalized between the minimum and maximum count for each clonal histogram.
