# Supplementary material for: Genetic Selection for Context-Dependent Stochastic Phenotypes: Sp1 and TATA Mutations Increase Phenotypic Noise in HIV-1 Gene Expression
Source: PLoS Comput Biol. 2013 Jul 11;9(7):e1003135. doi: 10.1371/journal.pcbi.1003135 (PMC3708878; doi:10.1371/journal.pcbi.1003135)
Supplement: Figure S3 — Log-binned histograms of clones infected with the WT Tat-null vector and the Sp1 mutant Tat-null vector. Clonal distributions were monomodal and wide with highly skewed distributions, which becomes apparent upon transformation to a real fluorescence axis. (PDF) [file pcbi.1003135.s003.pdf]

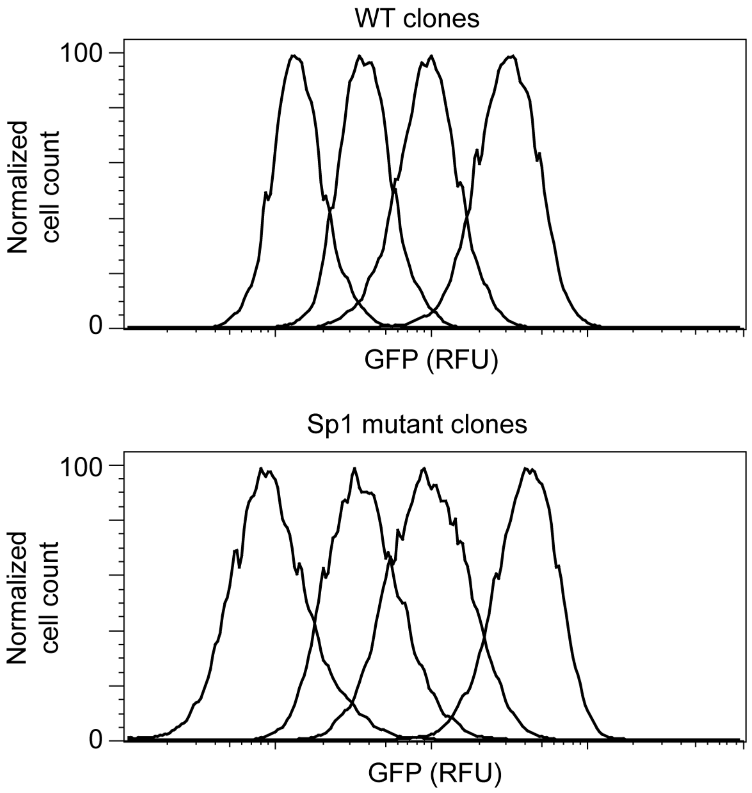

Figure S3. Log-binned histograms of clones infected with the WT Tat-null vector and the Sp1 mutant Tat-null vector. Clonal distributions were monomodal and wide with highly skewed distributions, which becomes apparent upon transformation to a real fluorescence axis.
