# Supplementary material for: Genetic Selection for Context-Dependent Stochastic Phenotypes: Sp1 and TATA Mutations Increase Phenotypic Noise in HIV-1 Gene Expression
Source: PLoS Comput Biol. 2013 Jul 11;9(7):e1003135. doi: 10.1371/journal.pcbi.1003135 (PMC3708878; doi:10.1371/journal.pcbi.1003135)
Supplement: Table S1 — Features used in cluster-based analysis of clonal populations (PDF) [file pcbi.1003135.s004.pdf]

Table S1: Features used in cluster-based analysis of clonal populations

**Feature**	**Reason**	**Additional notes on calculation**
1. Median	Measure of distribution center	
2. Inter-quartile range (IQR)	Measure of distribution width	
3. 3rd central moment	Measure of distribution skew	The cube root was taken
4. 4th central moment	Measure of kurtosis	The 4th root was taken
5. Peak separation	Measure of bimodality	Calculated based on smoothed histograms; equal to 0 for mono-modal distributions
6. Dip	Measure of bimodality	Equal to the difference between the normalized smoothed histogram maximum and the central minimum between the distribution peaks for bimodal distributions; 0 for mono-modal distributions
7. Dim weight	To distinguish Dim and Bright phenotypes	Operationally defined as the area under the normalized log-binned histogram to the left of cytometer bin 320 out of 1024 (covers the majority of the 'Dim' peak of the bulk multi-integration population for the WT transactivation circuit, as well as our auto-fluorescence profile)
8. Product of Dim weight and Bright weight	Measure of clonal distributions that include cells in both fluorescence ranges	Operationally defined as the distribution weight to the right of cytometer bin 512 out of 1024, which covers the majority of the 'Bright' peak of the bulk multi-integration distribution for the WT transactivation circuit, and is well-separated from the 'Dim' peak
