# Supplementary material for: Genetic Selection for Context-Dependent Stochastic Phenotypes: Sp1 and TATA Mutations Increase Phenotypic Noise in HIV-1 Gene Expression
Source: PLoS Comput Biol. 2013 Jul 11;9(7):e1003135. doi: 10.1371/journal.pcbi.1003135 (PMC3708878; doi:10.1371/journal.pcbi.1003135)
Supplement: Table S2 — Positions in the LTR with significant mutation rates following selection. (PDF) [file pcbi.1003135.s005.pdf]

**Table S2:** Summary of promoter positions with significant mutation rates following both selection screens[a]

| Position | Site | WT Base | # Mutations (Selected library) | | Expected # Mutations[b] (Unselected library) | | $Log_{10}$(p-value) (pairwise)[c] | | $Log_{10}$(Min p-value$^2$)[d] |
|---|---|---|---|---|---|---|---|---|---|
| | | | Activate | Deactivate | Activate | Deactivate | Activate | Deactivate | Combined |
| 380 | Sp1 III, p4 | G | 15 | 10 | 0.225 | 0.235 | <-25 | -12.7 | -25.5 |
| 426 | TATA, p2 | A | 9 | 6 | 0.917 | 0.958 | -6.29 | -3.28 | -6.56 |
| 412 | Core (n.i.[e]) | C | 3 | 19 | 0.173 | 0.179 | -3.10 | <-25 | -6.20 |
| 382 | Sp1 III, p6 | G | 10 | 3 | 0.218 | 0.235 | -13.1 | -2.72 | -5.43 |
| 391 | Sp1 II, p4 | G | 3 | 3 | 0.225 | 0.235 | -2.77 | -2.72 | -5.43 |
| 192 | U3 (n.i.) | A | 5 | 17 | 0.917 | 0.958 | -2.54 | -15.5 | -5.07 |
| 431 | TATA, p7 | A | 5 | 5 | 0.917 | 0.958 | -2.54 | -2.45 | -4.90 |

a. *Mutated positions were considered significant if both pairwise p-values < -2 ($p < 0.01$) and the $\log_{10}$(min p-value$^2$) < -4.*
b. *Expected mutation rate is base-type specific but not position-specific in the initial library.*
c. *Pairwise comparison of mutation rates between an individual screen and the unselected library*
d. *$Log_{10}$ of the square of the minimum of the pairwise p-values, which is a measure of the probability of the weaker screen giving that p-value or better, under the null hypothesis that the all 3 rates are the same.*
e. *n.i. = Not identified*
