# Supplementary material for: Genetic Selection for Context-Dependent Stochastic Phenotypes: Sp1 and TATA Mutations Increase Phenotypic Noise in HIV-1 Gene Expression
Source: PLoS Comput Biol. 2013 Jul 11;9(7):e1003135. doi: 10.1371/journal.pcbi.1003135 (PMC3708878; doi:10.1371/journal.pcbi.1003135)
Supplement: Table S3 — Point mutations introduced into LTR promoters used in our experimental studies. (PDF) [file pcbi.1003135.s006.pdf]

**Table S3**: Summary of LTR single point mutations

| Mutant Name | WT TFBS sequence[1] | Mutation |
|---|---|---|
| Sp1 site III (S3) | GAG**G**CGTGGC | G to A |
| TB position 2 | C**A**TATAAG | A to G |

*1. Mutated positions are in bold*
