# Supplementary material for: Genetic Selection for Context-Dependent Stochastic Phenotypes: Sp1 and TATA Mutations Increase Phenotypic Noise in HIV-1 Gene Expression
Source: PLoS Comput Biol. 2013 Jul 11;9(7):e1003135. doi: 10.1371/journal.pcbi.1003135 (PMC3708878; doi:10.1371/journal.pcbi.1003135)
Supplement: Table S4 — Sequences of HIV genes up to and including stop codons used in the sLTR-Tat-GFP vector. (DOCX) [file pcbi.1003135.s007.docx]

**Table S4**: HIV gene sequences with stop codons used in sLTR-Tat-GFP vector

| **Gene** | **Sequence from start codon to early stop codons** |
| --- | --- |
| *gag/pol* | ATGGGTGCGAGAGCGTCGGTATTAAGCGGGGGAGAATTAGATAAATGGGAAAA  AATTCGGTTAAGGCCAGGGGGAAAGAAACAATATAAA**TAATAA** |
| *env* | ATGAGAGTGAAGGAGAAGTATCAGCACTTGTGGAGATGGGGGTGGAAATGGGG  CACCATGCTCCTTGGGATATTGATGATCTGTAGTGCT**TAATAA** |
| *rev* | ATGGCAGGAAGAAGCGGAGACAGCGACGAAGAGCTCATCAGAACAGTCAGACTC  ATCAAGCTTCTCTATC**TAA** |
| *vif* | ATGGAAAACAGATGGCAGGTGATGATTGTGTGGCAAGTAGACAGGATGAGGATTA  ACACATGGAAAAGATTAGTAAAACACCAT**TAGTAA** |
| *vpr* | ATGGAACAAGCCCCAGAAGACCAAGGGCCACAGAGGGAGCCATACAATGAATGGA  CACTAGAG**TAA** |
| *vpu* | ATGCAACCTATAATAGTAGCAATAGTAGCATTAGTAGTAGCAATAATAATAGCAATA  GTTGTGTGGTCCATAGTAATCATA**TAATAA** |
