# Supplementary material for: Genetic Selection for Context-Dependent Stochastic Phenotypes: Sp1 and TATA Mutations Increase Phenotypic Noise in HIV-1 Gene Expression
Source: PLoS Comput Biol. 2013 Jul 11;9(7):e1003135. doi: 10.1371/journal.pcbi.1003135 (PMC3708878; doi:10.1371/journal.pcbi.1003135)
Supplement: Text S1 — Supporting computational methods. This text contains explanatory notes on feature-based clustering, estimates of Switching fraction, details of the Tat feedback model, including model equations and parameters. (PDF) [file pcbi.1003135.s008.pdf]

TEXT S1 – SUPPORTING COMPUTATIONAL METHODS

CONTENTS

I. Cluster-based criteria for identifying Switching distributions

II. Estimating Switching fractions from sub-sorted clonal data

III. A model of intrinsic expression variability for the Tat positive feedback circuit

IV. Model parameters

V. Model solution

VI. Time scales in the model

VII. Fitting Tat-null distributions

VIII. Estimating experimental sampling densities of basal transcription parameters over genomic integrations

IX. Phase diagrams-based calculation of Switching fractions

X. A model extension to include transcriptional reinitiation

I. Cluster-based criteria for identifying Switching distributions

Randomly selected clonal populations infected with single integrations of our experimental model of the HIV viral Tat-feedback circuit exhibit GFP distributions that fall into one of three categories: Dim, Bright, or Switching. These expression phenotypes are typically qualitatively distinguishable by eye. Here we discuss the qualitative features of these expression phenotypes that are apparent by eye, and our use of distribution clustering to define criteria for systematic phenotype assignment.

Clonal distributions that were qualitatively labeled as Dim (i.e., "by eye") approximately resemble those of the Tat-null system, which was analyzed in this study as well as in previous work (1, 2). These distributions are wide and highly right-skewed, but are mono-modal and far less variable than those categorized as Switching for the feedback circuit. Furthermore, Dim distributions typically demonstrate significant overlap with the Dim peak of their multi-integration parent bulk fluorescence distribution and with the autofluorescence distribution that was measured for uninfected cells, with a large majority of distribution weight falling in approximately the lower third of our cytometer log-fluorescence range. Clonal distributions that were qualitatively labeled as Bright were also mono-modal and similarly shaped, but with distribution peaks at higher fluorescence. These distributions are well separated from the Dim range of fluorescence, and instead demonstrate significant overlap with the Bright peak of their parent multi-integration bulk fluorescence distribution and minimal overlap with the autofluorescence distribution, with the large majority of distribution weight falling in the upper half of our log-fluorescence cytometer range.

In contrast to these Dim and Bright distributions, clonal distributions with significant weight in the middle of the log fluorescence range were typically significantly wider than Tat-null distributions, with significant distribution weight simultaneously in the Dim and Bright ranges of fluorescence. Many of these distributions demonstrated bimodality. It is these clonal distributions, with individual cells demonstrating both basal and transactivated expression, that we wished to label as Switching, and that we consider to be a model for stochastically-generated latent infection.

In previous studies, variegated phenotypes, such as 'Switching' distributions, had been scored by eye based on their distinctive features (1, 3). However, the range of expression phenotypes across a population of viral integrations is a continuum. Thus, there exist apparently 'Dim' distributions with tails that extend through the middle fluorescence range and apparently 'Bright' distributions with large opposite skew and tails extending across the middle fluorescence range, as well as distributions peaked in the middle 3rd of the log-fluorescence range that might be only slightly wider than typical Tat-null distributions but do not significantly extend into both the 'Dim' and 'Bright' fluorescence ranges.

In this study, our goal was to identify genetic elements that quantitatively modulate the distribution of expression phenotypes across a sampling of viral integrations. In particular, we were interested in identifying mutations that increase the Switching fraction. Thus, we required objective criteria for labeling phenotypes as Dim, Bright or Switching in order to quantitatively compare the distributions of phenotypes that arise under different experimental conditions. In particular, we needed to systematically extend our qualitative phenotypic scoring to those distributions whose phenotypes were ambiguous by eye. To solve this problem, we used feature-based clustering as a tool to identify by eye threshold values for distribution feature measures

that could be applied uniformly across the entire set of integration clones – a clonal distribution exceeding the cut-off value for any one of these feature measures was labeled as Switching. Our feature-based criteria, which we describe in more detail below, could be applied unambiguously to all of our clonal distributions, allowing us to quantitatively compare the distribution of phenotypes over viral integrations between mutant and WT Tat feedback circuits.

We began by identifying 8 distribution features, which distinctively characterized the clonal expression phenotypes that were unambiguously labeled by eye as Dim, Bright, or Switching (Table S1). All distribution features were calculated based on the log-binned relative-frequency fluorescence histograms generated by our cytometer, (covering 4 orders of magnitude in fluorescence on 1024 bins), and each feature was normalized by its inter-quartile range over the set of analyzed clones to put them on a comparable scales (except for Dim weight, which falls between 0 and 1 by design). See Figure S2A for the distribution of normalized features over the set of clonal distributions analyzed in this study.

In order to organize the full set of clonal distributions and view them simultaneously, we applied k-means clustering to the complete set of clonal expansions of single integrations of the transactivation circuit that were analyzed in this study (including all mutants and methods of selection). Clustering was implemented in MATLAB (The Mathworks), based on the 8 normalized distribution features enumerated above, using a Euclidean distance, on 20 clusters (for which distribution types that were qualitatively different by eye were well separated).

To use our clusters as a tool for systematic phenotypic determination, we considered 5 feature measures out of our group of 8 that most often by eye to identified Switching distributions: IQR (Feature 2); 3rd distribution moment (Feature 3); peak separation and dip for bimodal distributions (Features 5 and 6); and the product of Dim and Bright weights (Feature 8). We ordered our clusters by their centroid value separately for each of these features and chose as a cut-off value the cluster centroid such that the typical distribution shape in those clusters with centroid values above the cut-off appeared sufficiently variegated to be labeled as Switching by eye. Once these cut-offs were identified, we labeled a clonal distribution as Switching if any one of the considered 5 feature measures exceeded the identified cut-off, and imposed the additional requirement that all Switching distributions have weight in both the Dim and Bright fluorescence range (i.e. their value for Feature 8 was not 0). Distributions that were not labeled as Switching were then labeled as Bright if their 'Dim' weight (Feature 7) fell below 2/3, and the remaining distributions were labeled as Dim. All cut-offs are labeled in Figure S2A.

Figure S2B provides the full set of clustered distributions. Clusters are ordered there by the product of Dim and Bright weight (Feature 8) at the cluster centroid and by IQR (Feature 2) for those distributions with either Bright or Dim weight equal to 0. Because clustering was based on distribution features rather than the distributions themselves, the final clustering did not necessarily group distributions whose profiles coincided, but rather grouped distributions with similar shape features. For example, because only one of the eight features was a measure of center, similarly shaped distributions that were significantly shifted from one another on the log fluorescence scale with little overlap could be assigned to the same cluster. A heat-map presentation provides another visualization of the full set of clustered distributions (Figure S2C).

We find that clusters 9-20 (Figure S2B) are all highly variegated, and all distributions in these clusters are labeled as Switching. These distributions unambiguously depict the type of behavior that we envisioned as a Switching phenotype, and these distributions would have been

labeled as such by eye. Similarly, all distributions in clusters 1-4 are labeled as either Bright or Dim. These distributions are all mono-modal and more sharply peaked by comparison, and would not have been labeled as Switching by eye. Phenotypic labeling of distributions in the remaining clusters may have been ambiguous by eye, and it is here that our threshold-based criteria are an essential tool for systematic phenotype specification. It is on the basis of these systematic criteria that Switching fractions were calculated for the transactivation circuit variants analyzed in our study.

While our approach to phenotypic specification is not an unsupervised method for defining a Switching criterion, it provides a means of unambiguously extending our by-eye intuition as to which distributions are sufficiently variegated to merit this label. Our approach is further supported by our simulation analysis of the transactivation circuit, which suggests that the distribution shapes that we labeled as Switching are in fact associated with delayed switching between low and high levels of expression, as discussed in the main text.

II. Estimating Switching fractions from sub-sorted clonal data

A distinctive feature of the variegated phenotypes that are here labeled as Switching, is significant weight in the middle of the fluorescence range (which coincides with the dip in the multi-integration bulk-population histograms of the Tat feedback circuit). Here, as in previous work (1, 3), we found that collections of clonal populations generated from cells selected from the middle third of the bulk population fluorescence range were enriched in Switching phenotypes. Thus, sampling integrations of the Tat feedback circuit from the middle of the fluorescence range might provide a more efficient means of selecting and characterizing Switching phenotypes. However, our study required quantifying Switching fraction modulations over the full set of viral integrations for selected mutations, rather than the subset of integrations that are selected in a mid sort form the bulk population. Indeed, the sampling of integration clones induced by selecting cells only from the mid fluorescence range of the bulk population would be biased towards clones having more weight in the middle range of fluorescence (i.e. in the sort region). If our selected mutations were found to differentially affect the distribution of phenotypes for this mid-fluorescence-biased set of integrations, then effects on the Switching fraction of a mid sort might not be indicative of an effect on the Switching fraction for the full sampling of genomic integrations.

To overcome the above difficulty, we derived a method of estimating the Switching fraction over the full population of genomic integrations of the Tat feedback circuit, based on a sample of integration clones generated from mid-sorted cells. The derivation builds on an application of Bayes' Theorem, and requires an estimate of the ratio of bulk distribution weight in the sort region to the mean weight of the population of clonal Switching distributions in the sort region.

In the following, we are interested in the probability that a randomly selected cell taken from the bulk population of viral integrations will contain an integration of the transactivation circuit that would demonstrate a Switching phenotype by our categorization if that cell were expanded into a clonal population. We would like to relate this probability to the probability of selecting a Switching integration by choosing a cell whose fluorescence falls within a restricted range (the 'sort' region). This later probability is estimated by the fraction of Switching distributions measured experimentally for a set of clonal populations expanded from individual

cells selected from the sort region. For concreteness, we consider the Mid-range of fluorescence, as specified in our experiments, in our derivation.

To proceed, we assume that the bulk population of viral integrations consists of a representative sample of genomic integrations of the transactivation circuit. We further assume that the sub-population of cells generated from each integration (the descendants of each individual viral integration generated by the initial infection) is sufficiently randomized, such that the probability of observing a given range of fluorescence for any single cell in the bulk population is given by the weight in sort region of the steady-state distribution that would be observed if that cell were expanded to a large clonal population. Thus, we assume that the bulk fluorescence distribution that we measure samples from a steady-state distribution that is essentially a normalized weighted sum of the steady-state clonal fluorescence distributions generated by the full set of possible viral integrations; the weighting factor is proportional to the probability that a given viral integration is generated by the initial infections and subsequently observed in our experimental preparation.

Let S be the event that a randomly selected cell contains an integration that would be labeled as Switching if that cell were expanded to a clonal population. Let M be the event that the cell is found in the fluorescence range specified by a mid sort of the bulk population. Let $\sim M$ be the event that the selected cell is not found in the mid-sort region, i.e. the probability that its fluorescence falls outside of the mid-sort range. Then by the law of total probability,

$$P(S) = P(S|M)P(M) + P(S|\sim M)P(\sim M).$$

Here, $P(S|M)$ is the probability that the selected cell contains a Switching integration, given that its fluorescence was found to be in the fluorescence range of the mid sort. This conditional probability, of finding a Switching phenotype in a mid sort, is estimated by the fraction of the expanded clonal populations that were labeled as Switching out of the set of clonal populations that were expanded from the mid sort in a given experiment. Similarly, $P(M)$ is the probability of finding an infected cell with fluorescence in the range of the mid sort (with any integration phenotype) and is estimated by taking the weight of the bulk histogram in the mid-sort region (typically the sort region was defined to either include approximately the middle 1/3 or the middle 1/6 of the fluorescence range of the bulk population).

Unfortunately, we do not have a direct way to estimate $P(S|\sim M)$. This is the probability of finding a Switching integration outside of the mid-sort. Clones selected from this complement fluorescence range were only measured for our initial stratified estimate of the Switching fraction for the WT transactivation circuit (with probability sampling over the full range of fluorescence). This provides a way to validate the method derived here, and we found in the main text that the estimated Switching fractions match well (Figure 1D).

We proceed by way of Bayes' Theorem:

$$P(S|\sim M) = P(\sim M|S)P(S)/P(\sim M)$$

Here, $P(S)$ is precisely the Switching fraction that we wish to estimate (over the full population of genomic integrations), and $P(\sim M|S)$ is the weight of the fluorescence distribution of the full Switching population outside of the sort region, which may be written as:

$$P(\sim M|S) = 1 - P(M|S).$$

Combining this with our original expression, we find:

$$P(S) = P(S|M)P(M)/P(M|S),$$

which is simply another expression of Bayes Theorem. Thus, we require an estimate of the weight of the full Switching population in the sort region, $P(M|S)$. This probability is also equal to the average weight of a Switching clone in the sort region, taken over the full set of Switching integrations. We next proceed to obtain an estimate for this quantity.

By construction, all Switching distributions have weight in the mid-sort regions used in our experiments, because they have weight in both the Dim and Bright ranges and they are continuous. Thus, we can consider the set of Switching integrations, obtained from a mid sort, as a probability sample taken from the full set of Switching integrations. The sampling weight for each individual Switching integration is proportional to its weight in the sort region.

Let the random variable W_S represent the fluorescence distribution weight in the sort region of a randomly selected clone from the *full population* of Switching integrations, with associated probability density function (PDF) $\rho_S(w)$. Let W_M represent the distribution weight in the sort region of a randomly selected clone from a *mid-sorted population* of Switching integrations, with associated PDF, $\rho_M(w)$. If all Switching clones have some weight in the sort region (as is ensured by our Switching criteria), the PDF for the mid-sorted clones is given by:

$$\rho_M(w) = w\rho_S(w)/E[W_S]$$

Here, the probability of observing a weight w in the sort region for a cell selected from the sort region is proportional to that weight, and the expectation, $E_S[W] \equiv \int_0^1 \rho_S(w)dw = P(M|S)$, which acts as normalization, is precisely the probability that we seek to estimate. However, we require an estimate that is based only on a sample selected from the sort region, and we can only directly estimate the density $\rho_M(w)$ from such a sample, rather than $\rho_S(w)$. To solve this problem, we integrate the above expression and solve for $E[W_S]$. We find:

$$E[W_S] = \left(\int_0^1 (1/w)\rho_M\, dw\right)^{-1} \equiv 1/E[1/W_M]$$

Because the ordinary plug-in estimate for expectation on the right hand side above will not be robust to sampling error at small values of w and to experimental errors in these measurements, we considered estimates based on approximating the PDF by a Fourier series (coefficients matched to the first 4 distribution moments), or parametrically using a truncated Beta distribution. However, for our data set, we found all of these methods to provide estimates comparable to the plug-in value (with comparable confidence intervals, as calculated using a boot-strap approach), and therefore used the ordinary plug-in estimate for our analysis:

$$\hat{P}(M|S) = \hat{E}[1/W_M] = \left(\sum_{i=1}^{n_s} 1/w_i\right)/n_s$$

where the w_i are the mid-weights of the n_s Switching distributions obtained from our sample of expanded populations taken from the mid sort.

Finally, we used our above estimate of the average weight in the sort region for the Switching population ($P(M|S)$), together with our estimate of expected bulk weight in the sort region ($P(M)$), and our estimate of expected 'Switching' fraction for the mid sort ($\hat{P}(S|M) = n_s / n$ where n is the number of expanded clonal populations sampled in the mid sort), to obtain an estimate of Switching fraction over the full set of viral integrations in the bulk population. We calculated uncertainties about this point estimate, due to both errors in estimating the Switching fraction of our mid sort based on our sample and in using this to infer Switching fractions over the full set of viral integrations by the method outlined above, via a bootstrap method. This allowed a bootstrap estimated 95% confidence interval about our point estimate Switching fractions, as well 95% CIs for estimated differences between WT Switching fractions and mutant Switching fractions, which are quoted in the main text.

III. A model of intrinsic expression variability for the Tat positive feedback circuit

Our model of the Tat positive feedback circuit is an expansion of a basic Markovian model of gene expression that has been used in a number of other studies to analyze expression variability from promoters in the absence of feedback regulation (2, 4-6). Each process – gene activation, transcription, and translation – is assumed Markovian, proceeding at a fixed probability per unit time, which depends only on the current state of the system. The state of the system is specified by the ordered triple (*a*/*i*, *m*, *n*), representing the activation state of the promoter (*a* for active, *i* for inactive), the number of copies of the transcript of interest in the cell (*m*), and the number of copies of the protein of interest (*n*). For the transactivation circuit, we take the expressed protein to be Tat. The probability of finding the cell in a given state, will be denoted as $\phi(a/i,m,n)$.

The following elementary reactions define our model:

1) $I \xrightarrow{k_{a0}\left(1+\alpha_a f([P])\right)} A$ (gene activation, including Tat-dependence)

2) $A \xrightarrow{k_i} I$ (gene inactivation)

3) $A \xrightarrow{k_{t0}^{+}\left(1+\alpha_t f([P])\right)} A + T$ (transcript production, including Tat dependence)

4) $T \xrightarrow{k_t^{-}} X$ (transcript degradation, assumed to occur independently for each transcript)

5) $T \xrightarrow{k_p^{+}} T + P$ (protein production, assumed to occur independently for each transcript)

6) $P \xrightarrow{k_p^{-}} X$ (protein degradation, assumed to occur independently for each protein)

Here, A = active state of the gene, I = inactive state of the gene, T = transcript, P = protein, X = degraded. The above 'microscopic' rates represent probabilities per unit time for each reaction, and can be related to 'macroscopic' reaction rates via appropriate scaling by system volume.

The function, $f([P])$ characterizes transactivation in our model, where $[P] = n/v$ is the concentration of Tat (with cell volume v), which we assume to be proportional to GFP fluorescence. For $\alpha_a = \alpha_t = 0$, the model reduces to the previously considered model of stochastic expression from an unregulated promoter, considered here as a model for expression from our Tat-null system, which was analyzed in earlier work (2). To describe the positive feedback that characterizes the transactivation circuit, we considered a Michaelis-Menten form for $f([P])$, which specifies a linear rate enhancement with saturation:

$$f([P]) = \frac{[P]}{[P] + \lambda}$$

Such a form for the Tat-dependence of gene activation and transcription in our system could arise as described below.

Consider an expansion of our 2-gene-state model to include both Tat-bound and Tat-unbound forms of the active and inactive gene state (i.e. a 4-state gene model). Let the transition rate from the inactive to active state be $\kappa_{a,Tat}$ if Tat is bound and κ_{a0} if Tat is not bound, and let the transition rate back to the inactive state (κ_i) be unaffected by Tat binding. Let the rate of transcription from the Tat-bound active state of the promoter be $\kappa_{t,Tat}^{+}$, and let the transcription rate be κ_{t0}^{+} otherwise. Let Tat binding and unbinding occur also with linear kinetics, proportional to Tat concentration. Now, assume that the rate of binding and unbinding is much faster than the gene activation and inactivation rate (in particular, binding and unbinding is much faster than the transcriptional burst duration), such that the state of the gene effectively reaches equilibrium with respect to Tat binding between gene-state transitions, with equilibrium constant $1/\lambda$. The above form for the Tat-dependent gene activation and active-state transcription rates then results, and describes the effects of feedback in that transactivation circuit. The gene activation rate at full transactivation is given as $\kappa_{a,Tat} = \kappa_{a0}(1 + \alpha_a)$ and the active-sate transcription rate at full transactivation is given as $\kappa_{t,Tat}^{+} = \kappa_{t0}^{+}(1 + \alpha_t)$.

Based on the discussion above, the parameters that describe Tat feedback in our model of the transactivation circuit are as follows. 1) The fold-amplification of the gene activation rate at full transactivation, specified by $(1 + \alpha_a)$, which is also the fold-amplification of the transcripional burst frequency at full transactivation over the basal frequency in the bursting regime of the model. 2) The fold-amplification of the active-sate transcription rate at full transactivation, specified by $(1 + \alpha_t)$, which is also the fold-amplification of the transcriptional burst size at full transactivation over the basal bust size in the bursting regime of the model. 3) λ, which species the Tat concentration at which Tat is bound to the promoter half the time and the feedback begins to saturate. We note that the Tat-dependence for each type of feedback effect considered in our model (feedback to gene activation and to transcription) likely occur through different mechanisms, and could each saturate at a different Tat concentration. For simplicity, we

have only considered a single Tat dependence for the gene-activation and transcription rates, and assumed that the Tat dependence saturates at the same Tat concentration for both feedbacks.

IV. Model parameters

We analyzed the behaviors of our model at different levels of its biochemical parameters by generating a series of phase diagrams, as described in the main text and discussed further below. In this way, the model parameters that specify basal transcriptional dynamics were systematically varied over the range observed experimentally, as discussed in our earlier work (2), while feedback parameters, which are not well-characterized experimentally, were sampled discretely over a range of values. Feedback parameters used for the phase diagrams in the main text were selected to approximately match model predicted WT Switching fractions and Bright fractions (discussed further below), and to illustrate their variation.

The 3 model parameters characterizing Tat feedback (discussed above) were systematically sampled to generate a series of phase diagrams. Considered values for the fold-amplification for the transcription rate $\left(1+\alpha_t\right)$, which amplifies the transcriptional burst size, were 10, 15, 20, 22, 25, 30, 40; the WT value used in Figure 7A (main text) was 30, and the value used to demonstrate decreased amplification in Figure 7A was 20. Considered values for the fold-amplification for the gene-activation rate $\left(1+\alpha_a\right)$, which amplifies the transcriptional burst frequency, were 1, 4, 7, 10; the value used for all phase diagrams in the main text was 7. These amplification factors combine approximately multiplicatively in their effect on mean expression, so that the fold amplification of mean expression at saturating Tat, relative to no Tat (basal), ranged from 40 to 400 over the series of phase diagrams that was analyzed. Actual amplification factors for mean expression were typically smaller than these values in practice because for basal expression patterns low levels of Tat expression still drove expression levels slightly above the level expected if the feedback were disabled (the Tat-null system), and because transactivated expression does not fully saturate Tat binding. We also considered values of the feedback saturation parameter corresponding to the low end and high end of the mid-fluorescence range of our bulk-population histograms; the lower value was used for the phase diagrams in the main text, corresponding to a protein number of $60\kappa_p^+/\kappa_p^-$.

For each of these combinations of transactivation parameters, the parameters describing basal transcription in our model (transcriptional burst size and burst frequency) were systematically varied over the range observed in our analysis of Tat-null clonal distributions (Figure 6, main text). In addition, adjustments were made in our sampling of basal transcriptional burst sizes to ensure that for each basal burst frequency considered in each phase diagram, basal burst sizes covering the range of values leading from Dim to Bright phenotypes were sampled. To generate a single phase diagram, at least 8 basal burst frequencies we sampled, and for each, at least 20 basal burst sizes were sampled, with parameter sampling becoming finer near phenotypic boundaries. Remaining model parameters we fixed at previously calibrated values, with $\kappa_i = 20\kappa_t^-$ ensuring that transcriptional bursts are relatively short, as described (2).

Transcriptional dynamics in the bursting regime ($\kappa_i \gg \kappa_t^-, \kappa_t^+/\kappa_i$ of order 1 or greater) were always found to give the best account for Tat-null distributions (discussed further below),

and we assumed for our transactivation model that a bursting dynamic is maintained in the presence of Tat. In particular, the fact that $\kappa_i = 20\kappa_t^-$ is independent of Tat binding in the model, and that transcription rates only increase with Tat binding, ensures that transcription always occurs in bursts at any concentration of Tat in the model. Thus, transcript production in our model always occurs in bursts, with the transcriptional burst size and frequency increasing in the presence of Tat.

V. Model solution

The Kolmogorov system of equations, known as the chemical master equation for chemically reacting systems such as our model, specifies the evolution of the probability distribution for our model, as follows:

$$\frac{d\phi(a,m,n)}{dt} = -\left(\kappa_i + k_{t0}^+\left(1+\alpha_t f(n/\nu)\right) + mk_t^- + mk_p^+ + nk_p^-\right)\phi(a,m,n) + k_{a0}\left(1+\alpha_a f(n/\nu)\right)\phi(i,m,n)$$
$$+k_t^+\left(1+\alpha_t f(n/\nu)\right)\phi(a,m-1,n) + (m+1)k_t^-\phi(a,m+1,n) + mk_p^+\phi(a,m,n-1) + (n+1)k_p^-\phi(a,m,n+1)$$

$$\frac{d\phi(i,m,n)}{dt} = -\left(k_{a0}\left(1+\alpha_a f(n/\nu)\right) + mk_t^- + mk_p^+ + nk_p^-\right)\phi(i,m,n) + \kappa_i\phi(a,m,n)$$
$$+(m+1)k_t^-\phi(i,m+1,n) + mk_p^+\phi(i,m,n-1) + (n+1)k_p^-\phi(i,m,n+1)$$

This model does not admit an analytic solution, nor is it even possible to analytically calculate distributions moments – distribution moments of all orders are analytically calculable for the model in the absence of feedback, but here each distribution moment is coupled to higher moments and the system of equations can only be closed by methods of moment completion that approximate higher moments. Stochastic simulation could be used to sample model trajectories and approximate probability distributions for finding the system in any combination of gene state, transcript number, and protein number (and should make use of approximations for the large numbers of protein molecules that would be present in a typical cell (7)). However, we preferred to solve the master equation numerically, as a more efficient and accurate approach, given the small numbers of reacting species, following our approach in earlier work (2). Briefly: the system was truncated at sufficiently large protein and transcript numbers, which are effectively 'almost never' sampled by the system; states with large transcript and protein numbers were grouped with neighboring states in a coarse-graining approach; and transition rates between these grouped states were approximated by interpolation. The resulting system of linear ODEs effectively interpolate between a numerical approximation to the Fokker-Plank equation for the system at large protein and transcript numbers, and the exact master equation at smaller molecular numbers.

The system was numerically integrated semi-implicitly to ensure stability (8), until the distribution was approximately stationary (see Skupsky et al. (2) for further discussion). Additional care was taken to ensure that steady state was reached for our transactivation model, because bimodal distributions exist for some combinations of model parameters, with delayed switching between Dim and Bright expression states (i.e. these are the Switching distributions that are the focus of our present study). For this reason, to obtain steady-state distributions of the transactivation model, each simulation was initialized first in a Dim state (the steady-state

probability distribution obtained for the smallest combination of basal transcriptional burst size and frequency considered in each phase diagram was used for this initialization), and evolved until the protein distribution became stationary. The system was then initialized in a Bright transactivated state (the system steady-state probability distribution obtained for the largest combination of basal transcriptional burst size and frequency considered in each phase diagram was used for this initialization) and evolved again until the protein distribution became stationary. The system was evolved for sufficient time, such that the stationary distributions for the two initializations matched to a pre-specified accuracy.

VI. Time scales in the model

In earlier work (2), we had estimated a protein dilution time of approximately 20h ($1/\kappa_p^-$) and a transcript decay time of approximately 5h ($1/\kappa_t^-$) for our system. Typical basal transcriptional burst frequencies were found on the order of one per transcript decay time for the Tat-null model (Figure 6, main text), and burst frequencies increase with Tat concentration for the transactivation model. Thus, because the protein decay time is the longest time scale in our model, we expect this time scale to approximately determine the time scale of distribution equilibration for single-peaked expression phenotypes in our model. This means that, because typical burst frequencies are of the same order as the transcript decay rate, mono-modal expression phenotypes equilibrate on the time scale of several basal transcriptional bursts. On the other hand, for combinations of model parameters that lead to highly variegated phenotypes, where steady-state distributions include both cells demonstrating basal expression levels and cells demonstrating highly transactivated expression, the longer time-scale of transitions between these two expression regimes will determine distribution equilibration times. These transitions will depend on the infrequent occurrence of multiple larger and more frequent transcriptional bursts for activation, and multiple smaller and less frequent transcriptional bursts for deactivation. Thus, we find that the parameter regimes that specify Switching phenotypes in our model demonstrate significant delays in equilibration and approach to steady state. These time scales ranged from days to weeks in our simulations, as discussed further below.

We quantified delayed activation in our model as follows. The model was initialized in a basal expression state, corresponding to the lowest combination of basal burst size and frequency sampled in each phase diagram. The distribution of first-passage times was then calculated for cells crossing a threshold fluorescence value by imposing an absorbing boundary at this protein number in our simulations (the value of protein number that was used as a threshold corresponds to the cytometer fluorescence specifying the boundary of the Dim expression range for our feature-based clustering of clonal distributions). The time at which half of the cells had crossed this threshold was recorded as the Switching time in our model analysis. If this condition did not occur after a simulation time corresponding to approximately 400 hours of real time, it was considered that the distribution remained Dim and never transactivated.

Using the above quantification of equilibration time, parameter combinations specifying Bright phenotypes resulted in Switching times of order one or a few days, corresponding to several protein decay times, as expected. On the other hand, parameter combinations that specified Switching distributions by our feature-based criteria demonstrated equilibration times of order many days to weeks, as discussed in Figure 2C (main text).

Our quantification of equilibration time, and its relevance to the expression phenotypes in our system, is further motivated as follows. For the Switching phenotype to serve as a model for latency, we require an expression phenotype that will not transactivate during the active time of a T-cell (a primary source of latent infections), before it has transitioned to a memory state. On the other hand, if a latently infected cell is to spread the infection when it reactivates, it must do so within the lifetime of the T-cell after it reactivates from the memory state. If we make the simplifying assumption that these two times are the same and that the state of the reactivated cell is comparable to the initially infected cell, then the probability of significant viral expression in a cell before it transitions to the memory state is the same as for the reactivated T-cell, over the same time interval. If this probability is denoted p, then the probability that a cell does not significantly express viral proteins during the time before transition to the memory state (this is thought to be a requirement for the transition to occur), but does express the virus during the lifetime of the reactivated T-cell (otherwise the integrated virus is harmless) is $p(1-p)$. This probability is maximized at $p = 1/2$. Thus, the equilibration time that we have recorded from our simulations specifies the memory-state transition time and reactivated host-cell lifetime for which a given viral integration is optimized to produce a latent infection that is capable of reactivation and viral spread. In particular, we note that equilibration times from days to weeks are predicted by the mode for Switching phenotypes, and thus viral integrations that optimally produce latent-but-reactivatable infections exist for any memory-state transition time and reactivated host-cell life time in this range.

While the simplifying assumptions considered here may not hold for *in vivo* infections, a similar analysis could be carried out to determine the optimal reactivation time for an in-vivo viral integration to specify a latent-but-reactivatable infection, based on a more detailed model. Thus, the discussion here is meant to give an intuitive interpretation to model-based equilibration times that we have calculated and to demonstrate its relevance to a model of stochastically generated latent infection. The model-based equilibration times that we have calculated provide an important link between the steady-state expression phenotypes that we have measured in our experiments, and the dynamics of switching between expression states, which are the hallmarks of a Switching phenotype. For this reason, we consider the Switching phenotype to be a model for stochastically generated latent viral infection, for which we designed our experimental screen to select.

VII. Fitting Tat-null distributions

The Tat-null model fixes $\alpha_a = \alpha_t = 0$, and is the same model that was fit to protein expression distributions from a similar model viral system in earlier work (2). We followed a slight modification of the procedure in that study. Here, rather than fitting the full fluorescence histogram for each distributions, we fit the first 8 central moments of each clonal fluorescence distribution, which were estimated from each fluorescence histogram after autofluorescence correction. Fit parameters were: the gene activation and inactivation rate, and the active-state transcription rate (for steady state distributions, the transcript decay rate can be effectively scaled to 1, so that the remaining model parameters are all measured relative to the transcript decay rate). However, it was always found that the best model fits were in the transcriptional bursting regime. In this regime, the gene inactivation rate is set sufficiently high, it does not affect

distribution fits (we chose $\kappa_i = 20\kappa_t^-$, following our earlier work), and the model could be effectively parameterized by basal transcriptional burst size ($b = \kappa_{t0}^+ / \kappa_i$) and frequency ($\kappa_{a0}$). For each combination of model parameters, the first 8 central moments of the protein distribution were calculated analytically (2), and scaled to linearly convert from protein number to cytometer RFU for comparison to the experimental data. An initial-guess transcriptional burst size and burst frequency was calculated analytically, based on the mean and variance of each experimental fluorescence distribution. Then, a non-linear minimization routine was used to find the best-fit transcriptional burst size and burst frequency that minimized the sum of squared relative deviation between the first 8 central moments of the experimental and model fluorescence distributions. The best-fit values were generally close to the initial guess values.

To confirm that the best-fit model parameters for each clonal distribution were indeed in the transcriptional bursting regime for each of our experimental Tat-null vectors, we again followed the approach of Skupsky et al (2). For each clone, we scanned through fixed values of the gene inactivation rate, κ_i. Consistent with this earlier work, we always found the optimal fits were at the largest values of κ_i, corresponding to the shortest active-state durations ($\tau = 1/\kappa_i$). Thus, the best model fits for the Tat-null distributions were always in the transcriptional bursting regime.

VIII. Estimating experimental sampling densities of basal transcription parameters

Our model phase diagrams tell us, for each combination of transactivation parameters, which combinations of basal transcriptional burst sizes and burst frequencies lead to each type of expression phenotype at steady state. In order to use our model to estimate the fraction of viral integrations that specify a Switching phenotype, we therefore required an estimate from our experiments of the probability density with which the virus samples basal transcriptional burst sizes and burst frequencies through its sampling of genomic environments, as dictated by random integration upon infection, for each viral vector that we analyzed. We obtained these estimates by combining information from our model fits of Tat-null clonal distributions and the corresponding bulk-integration distributions (Figure 6, main text), as described below.

Our model fits of the sampled Tat-null clonal distributions provide best-fit basal transcriptional burst sizes and frequencies. To quantify the variation of transcriptional burst size and frequency over integration positions for each vector, these quantities were regressed against clonal expression means in a log-log plot (Figure 6C-D, main text). These regressions provide an estimate of the experimental viral sampling densities of transcriptional burst size and frequency over integration positions for each vector, at fixed values of expression mean (that is, conditional distributions on expression mean). However, these regressions do not provide any information about the sampling of expression means over viral integration positions, because expression mean was used to select the single-integration clones that were analyzed. Specifically, cells were selected for clonal expansion in our Tat-null system from the approximately the brightest 20% of the Tat-null bulk-integration population for each vector to ensure sufficient expression for auto-fluorescence deconvolution and model fitting. Thus, the distribution of clonal expression means in our sample of single-integration Tat-null clones does not provide an estimate of the distribution of expression means over the full set of Tat-null genomic integrations.

To obtain an estimate of the sampling density of basal expression means over viral integrations, we used the bulk-integration histograms for our Tat-null vectors (Figure 6A, main text), after smoothing and autofluorescence deconvolution. By combining the information in our Tat-null bulk-integration histograms with our regression of log best-fit transcriptional burst frequency against log expression mean from our sampled Tat-null integration clones, we obtained an estimate the sampling density of basal transcriptional burst size and burst frequency over the full set of genomic integrations sampled in our experiments, as discussed below.

We have the following identity for the joint probability of obtaining a viral integration leading to a mean protein expression within a range $d\mu$ about a value μ, and a transcriptional burst frequency within a range $d\kappa_a$ about a value κ_a:

$$P(\kappa_a,\mu)d\mu d\kappa_a = P(\mu)P(\kappa_a|\mu)d\mu d\kappa_a$$

The corresponding probability densities can be written as:

$$f(\kappa_a,\mu) = g(\mu)h(\kappa_a|\mu)$$

Based on our regression analysis, we can approximate the density of transcriptional burst frequency, conditional on expression mean, as

$$h(\kappa_a|\mu) = h_l(\log(\kappa_a)|\mu)\frac{d(\log(\kappa_a))}{d\kappa_a} = \frac{N(\beta_0 + \beta_1\log(\mu),S)}{\kappa_a}$$

Here, $h_l(\log(\kappa_a)|\mu)$ is the conditional density for log transcriptional burst frequency, which is related to $h(\kappa_a|\mu)$ via the above change-of-variable formula for probability densities; $N(a,b)$ is the normal probability density with mean *a* and standard deviation *b*; β_0 and β_1 are regression coefficients for the log-log regression of clonal transcriptional burst frequency against clonal expression mean, and S is the standard estimate of the standard deviation of the random component of the normal regression model, calculated based on the residuals of our regression.

Next, we estimated the density $g(\mu)$ by deconvolving from our bulk fluorescence distribution a 'typical' clonal fluorescence distribution of our Tat-null system, which was specified as follows. Earlier work (2), as well as our current analysis, indicated that clonal Tat-null distributions in our system demonstrate a characteristic shape variation over integration positions, with the distribution variance proportional to the mean raised to a power slightly less than 2. To a good approximation, these distributions maintain a characteristic scale on a log fluorescence scale that is approximately normal (i.e. on a linear scale, these distributions are approximately log-normal). In particular, by scaling the fluorescence of each of our Tat-null distributions such that each distribution was shifted to a common mean for each experimental vector, we found that the distributions super-impose nicely. We averaged these shifted densities to obtain a 'typical' Tat-null clonal distribution, which approximates the distribution about any mean log fluorescence value for a given Tat-null clone. We denote this 'typical' distribution by $\varphi(x_l - \mu_l)$, where $x_l = \log(x)$ is the log cellular fluorescence and μ_l is the mean log fluorescence of the distribution. Then our bulk log fluorescence distribution, which we denote by $B(x_l)$, can be considered as a convolution of the distribution of clonal mean log-fluorescences

over integration positions, which we denote as $g_l(\mu_l)$, and the 'typical' distribution log-fluorescence distribution $\varphi(x_l - \mu_l)$. We thus estimated $g_l(\mu_l)$ by first smoothing our experimental log-binned bulk fluorescence histograms to obtain $B(x_l)$, and then performing the deconvolution by standard methods (in Fourier space, using a Weiner filter). Because the log-binned bulk distributions in our study are much wider than the 'typical' clonal distribution for each experimental vector, the shape of $g_l(\mu_l)$ was very similar to $B(x_l)$, and in particular was insensitive to modifications in the shape of the 'typical' Tat-null clonal distribution that was used in the deconvolution.

Next, the sampling density of clonal expression mean fluorescence, $g(\mu)$, was obtained from the estimated sampling density of mean log fluorescence $g_l(\mu_l)$ via a simple change of variables as $g(\mu) = g_l(\mu_l(\mu))(d\mu_l / d\mu)$, and we assumed that μ_l is related to mean fluorescence by $\mu_x = e^{u_l + \sigma_l^2/2}$, as for a log-normal distribution. Here, σ_l^2 is the variance of our estimated 'typical' clonal log-fluorescence distribution. Lastly, the estimated density of fluorescence means was converted to a density in transcript mean according to $\mu = C\mu_x$ where C is a constant that had been calibrated following our approach in Skupsky et al. (2), and the relationship is expected to be linear for the Tat-null system.

Finally, the mean transcript number can be written in terms of transcriptional burst size and burst frequency in the bursting regime, according to $\mu = \kappa_a b$. Thus, we estimated the density $f(\kappa_a, \mu) = g(\mu)h(\kappa_a|\mu)$ using the above estimates of $h(\kappa_a|\mu)$ and $g(\mu)$, and used this to estimate a sampling density in terms of transcriptional burst size and frequency via one more final change of variables, according to $\tilde{f}(\kappa_a, b) = f(\kappa_a, \kappa_a b)\partial\mu / \partial b = \kappa_a f(\kappa_a, \kappa_a b)$. This estimated basal parameter sampling density was then used, in turn, to estimate model-predicted Switching fractions at fixed values of model feedback parameters, based on calculated phase diagrams, as outlined below.

IX. Phase diagrams-based calculation of Switching fractions

To generate model-predicted Switching fractions, we generated estimated basal parameter sampling densities for the WT and SP1 mutant experimental vectors that were analyzed in the main text, as described above. Then for each analyzed combination of transactivation parameters, a model-predicted Switching fraction was estimated for each vector by summing the estimated basal parameter distribution weight in the Switching region on the phase diagram. Similarly, Bright fractions could be calculated by summing the basal parameter distribution weights in the Bright region of each phase diagram. Phenotypic boundaries in all phase diagrams were similarly shaped for all analyzed sets of transactivation parameters that generated robust Switching phenotypes, though their positions and separation varied. This shifting of phenotypic boundaries with model feedback parameters could account for changes in phenotypic fractions, as described in the main text.

The transactivation parameters used in the main text were selected to specify a Switching fraction of approximately 10% and a Bright fraction of approximately 45% for our WT vector,

qualitatively matching our experimental data (Figure 7, main text). For these parameter values, the basal parameter sampling density estimated for the Sp1 mutant led to a predicted Switching fraction of approximately 20% and a Bright fraction of approximately 30% (Figure 7, main text). Though this enrichment is well below the experimentally observed value of nearly 6-fold enrichment, it does begin to approach the lower bound of the estimated 95% CI for our experiments. More importantly, it indicates that small effects on the basal parameter sampling density over a set of viral integrations can still significantly affect the Switching fraction that results in the presence of feedback. In addition, further effects on the basal parameter sampling density at low basal transcriptional burst sizes and frequencies, which were not well resolved in our analysis, could further contribute.

X. A model extension to include transcriptional reinitiation.

A natural extension of the two-state model of gene expression that we considered adds a third gene state to explicitly model the process of transcriptional reinitiation. In this extended model, the gene can be 1) in an inactive state that produces no transcript (*I*); activation (with rate k_a) then makes the gene accessible to RNA polymerase and transcription-complex binding, and transcription proceeds from 2) this transcriptionally active state (*A*) at rate k_t^+ as in the two-state model. Following each transcript-production event, the gene is left in 3) a refractory state (*R*), which produces no transcript; return to the active state (reinitiation) occurs at rate k_r; return to the inactive (inaccessible) state only occurs from the active state, with rate k_i as in the two-state model). The elementary reactions describing these gene-state transitions in the extended model, are as follows:

1) $I \xrightarrow{k_{a0}(1+\alpha_a f([P]))} A$ (gene activation, including Tat-dependence)

2) $A \xrightarrow{k_i} I$ (gene inactivation)

3) $A \xrightarrow{k_{t0}^+(1+\alpha_t f([P]))} R + T$ (transcript production, including Tat dependence, and the gene-state becomes refractory).

3.5) $R \xrightarrow{k_r} A$ (transcriptional reinitiation)

The remaining processes of transcript degradation, and protein production and degradation, proceed as in the 2-state model. The transcriptional behavior of such a cyclic three-state gene model has been analyzed by Tang et al. in the absence of feedback (9).

Our expanded model could be solved and analyzed by similar methods as those used here to solve our two-state model of the transactivation circuit. However, for the purposes of investigating whether such a model modification could account for the Switching-fraction enhancement seen in our experiments, we considered a simplification that maps the three-state model onto our two-state model through a redefinition of model parameters, based on the following argument.

The three-sate model remains Markovian. Therefore the time for each gene-state transition is still exponentially distributed. During a transcriptional burst (that is, during the time from a gene-activation event until the next inactivation event), the mean time between transcript production events is given as $\tau_t = 1/\kappa_t^+ + 1/\kappa_r$ (the sum of the average transcript production

time and the average reinitiation time). The time between transcript-production events will no longer be exponentially distributed (its distribution will be a convolution of the exponential distribution with rate κ_t^+ that describes the time till the next transcription event when the gene is in the active state and the exponential distribution with rate κ_r that describes the time until a reinitiation event when the gene is in the refractory state, leading to a gamma distribution when the two rates are equal). However, in the bursting regime, when a significant number of transcripts are produced during each transcriptional burst, burst-size variability occurs primarily due to variability in the length of each transcriptional burst (this time is also exponentially distributed, with rate κ_i), rather than due to variability in the time between each transcript-production event within a transcriptional burst. In other words, the distribution of times between transcript production events, which is the only feature of transcriptional dynamics that is affected by the addition of a reinitiation step to our model, will primarily affect the distribution of numbers of transcripts and proteins in the cell through its effect of the mean time between transcript production events, rather than through effects on variability in this time.

Thus, rather than explicitly modeling the three-state model, we considered the two-state model with a rescaled transcript production rate: $\kappa_t^* = 1/\tau_t = \kappa_r \kappa_t^+ / \left(\kappa_t^+ + \kappa_r\right)$. If we use the same form for the transcription rate in the presence of Tat that was used for our two-state model of the transactivation circuit, $\kappa_t^+ = \kappa_{t0}^+ \left(1 + \alpha_t f\left(\left[P\right]\right)\right)$ with $f\left(\left[P\right]\right) = \dfrac{\left[P\right]}{\left[P\right] + \lambda}$, then the transcription rate for the three-state-gene model of the transactivation circuit, which effectively includes reinitiation via the above-argued separation of time scales, can be written as:

$$\kappa_t^* = \kappa_{t0}^* \left(1 + \alpha_t^* f^*\left(\left[P\right]\right)\right)$$

The rescaled parameters defined here are related to those of the three-state model under the above assumption of separation of time scales, according to:

$$\kappa_{t0}^* = \kappa_{t0}^+ \frac{\kappa_r}{\kappa_{t0}^+ + \kappa_r} \text{ (rescaled basal transcription rate)}$$

$$\alpha_t^* = \alpha_t \frac{\kappa_r}{\kappa_{t0}^+ + \alpha_t \kappa_{t0}^+ + \kappa_r} \text{ (rescaled amplification factor for transactivated transcription rate)}$$

$$f^*\left(\left[P\right]\right) = \frac{\left[P\right]}{\left[P\right] + \gamma^*} \text{ (rescaled Tat-dependence for transactivated transcription)}$$

$$\gamma^* = \gamma \frac{\kappa_{t0}^+ + \kappa_r}{\kappa_{t0}^+ + \alpha \kappa_{t0}^+ + \kappa_r} \text{ (rescaled feedback saturation parameter)}$$

In other words, the model and its parametric dependencies has the same form as the two-sate model with the above parametric rescaling.

For fast reinitiation ($\kappa_r \gg \kappa_t^+$), the rescaled model parameters take their two-state-model values. On the other hand, for moderate values of the reinitiation rate ($\kappa_t^+ \sim \kappa_r \gg \kappa_{t0}^+$), including transcriptional reinitiation in the model has little effect on the basal transcription rate, but decreases the maximal fold-increase of transactivated expression over basal, causing the affect of Tat

feedback on its transcription rate to saturate earlier. In combination, these two effects specify a perturbation the decreases mean Bright expression but has little effect on initial transactivation dynamics from a Dim expression state. As a result, bright expression peaks are shifted towards lower fluorescence, but remain phenotypically Bright, and phenotypic boundaries are minimally affected, as illustrated in Figure 7A (main text). The phenotypic boundaries calculated for unimpaired reinitiation in the main text use $\kappa_r = 500\kappa_i$, which means that the reinitiation rate is equivalent to the transcript production rate that would generate transcriptional bursts of average size 500 transcripts if reinitiation were infinitely fast. Qualitative behaviors in our phase diagrams were preserved in our simulations as the reinitiation rate was decreased, until a value of approximately $\kappa_r = 20\kappa_i$. At these higher levels of reinitiation impairment, transactivated expression is sufficiently weakened, such that the variegated distributions that result do not demonstrate sufficient width to be labeled as Switching by our experimental criteria, and the Switching region is lost from the phase diagram. a value of $\kappa_r = 100\kappa_i$ was chosen to demonstrate the effects of moderate impairment in the main text, which shifts Bright expression peaks (Figure 7C, main text) but still approximately preserves the Switching region of the phase diagram (Figure 7A, main text).

References

1. Weinberger LS, Burnett JC, Toettcher JE, Arkin AP, & Schaffer DV (2005) Stochastic gene expression in a lentiviral positive-feedback loop: HIV-1 Tat fluctuations drive phenotypic diversity. (Translated from eng) *Cell* 122(2):169-182 (in eng).
2. Skupsky R, Burnett JC, Foley JE, Schaffer DV, & Arkin AP (2010) HIV promoter integration site primarily modulates transcriptional burst size rather than frequency. *PLoS Comput Biol* 6(9).
3. Burnett JC, Miller-Jensen K, Shah PS, Arkin AP, & Schaffer DV (2009) Control of stochastic gene expression by host factors at the HIV promoter. *PLoS Pathog* 5(1):e1000260.
4. Peccoud JaY, B. (1995) Markovian Modeling of Gene-Product Synthesis *Theor. Pop. Biol.* 48:13.
5. Raser JM & O'Shea EK (2004) Control of stochasticity in eukaryotic gene expression. (Translated from eng) *Science* 304(5678):1811-1814 (in eng).
6. Thattai M & van Oudenaarden A (2001) Intrinsic noise in gene regulatory networks. (Translated from eng) *Proc Natl Acad Sci U S A* 98(15):8614-8619 (in eng).
7. Gillespie DT (1977) Exact stochastic simulation of coupled chemical reactions. *Journal of Physical Chemistry* 81(25):2340-2361.
8. Press WHT, S.A.; Vetterling, W.T.; Flannery, B.P. (2007) *Numerical Recipes 3rd Edition: The Art of Scientific Computing* (CAmbridge University Press, New York).
9. Tang M (2009) The mean frequency of transcriptional bursting and its variation in single cells. (Translated from Eng) *J Math Biol* (in Eng).
